# Conjugation of Aminoadamantane and γ-Carboline Pharmacophores Gives Rise to Unexpected Properties of Multifunctional Ligands

**DOI:** 10.3390/molecules26185527

**Published:** 2021-09-11

**Authors:** Sergey O. Bachurin, Galina F. Makhaeva, Elena F. Shevtsova, Alexey Yu. Aksinenko, Vladimir V. Grigoriev, Pavel N. Shevtsov, Tatiana V. Goreva, Tatiana A. Epishina, Nadezhda V. Kovaleva, Elena A. Pushkareva, Natalia P. Boltneva, Sofya V. Lushchekina, Alexey V. Gabrelyan, Vladimir L. Zamoyski, Lyudmila G. Dubova, Elena V. Rudakova, Vladimir P. Fisenko, Elena V. Bovina, Rudy J. Richardson

**Affiliations:** 1 Institute of Physiologically Active Compounds, Russian Academy of Sciences, 142432 Chernogolovka, Russia; gmakh@ipac.ac.ru (G.F.M.); shevtsova@ipac.ac.ru (E.F.S.); alaks@ipac.ac.ru (A.Y.A.); grigor@ipac.ac.ru (V.V.G.); pnshevtsov@gmail.com (P.N.S.); tg1960@mail.ru (T.V.G.); tatiana.epishina@mail.ru (T.A.E.); kovalevanv@ipac.ac.ru (N.V.K.); l.kickeeva@yandex.ru (E.A.P.); boltneva@ipac.ac.ru (N.P.B.); sofya.lushchekina@gmail.com (S.V.L.); avg@ipac.ac.ru (A.V.G.); vzam@yandex.ru (V.L.Z.); dubova@ipac.ac.ru (L.G.D.); rudakova@ipac.ac.ru (E.V.R.); bovina_e@ipac.ac.ru (E.V.B.); 2 Emanuel Institute of Biochemical Physics, Russian Academy of Sciences, 119334 Moscow, Russia; 3 I.M.Sechenov First Moscow State Medical University, 8 Build. 2 Trubetskaya Str., 119991 Moscow, Russia; vpfisenko@mail.ru; 4 Department of Environmental Health Sciences, University of Michigan, Ann Arbor, MI 48109, USA; rjrich@umich.edu

**Keywords:** multifunctional agents, Alzheimer’s disease, cholinesterases, NMDA receptor, mitochondrial permeability transition (MPT) pore, microtubules

## Abstract

A new series of conjugates of aminoadamantane and γ-carboline, which are basic scaffolds of the known neuroactive agents, memantine and dimebon (Latrepirdine) was synthesized and characterized. Conjugates act simultaneously on several biological structures and processes involved in the pathogenesis of Alzheimer’s disease and some other neurodegenerative disorders. In particular, these compounds inhibit enzymes of the cholinesterase family, exhibiting higher inhibitory activity against butyrylcholinesterase (BChE), but having almost no effect on the activity of carboxylesterase (anti-target). The compounds serve as NMDA-subtype glutamate receptor ligands, show mitoprotective properties by preventing opening of the mitochondrial permeability transition (MPT) pore, and act as microtubule stabilizers, stimulating the polymerization of tubulin and microtubule-associated proteins. Structure–activity relationships were studied, with particular attention to the effect of the spacer on biological activity. The synthesized conjugates showed new properties compared to their prototypes (memantine and dimebon), including the ability to bind to the ifenprodil-binding site of the NMDA receptor and to occupy the peripheral anionic site of acetylcholinesterase (AChE), which indicates that these compounds can act as blockers of AChE-induced β-amyloid aggregation. These new attributes of the conjugates represent improvements to the pharmacological profiles of the separate components by conferring the potential to act as neuroprotectants and cognition enhancers with a multifunctional mode of action.

## 1. Introduction

Every year, nearly 10 million new patients are diagnosed with neurodegenerative diseases (NDs), such as Alzheimer’s disease (AD) and other types of dementias [1,2]. As of 2019, over 2100 clinical trials were performed with various agents as treatments for AD [3]. However, there are only a few drugs recommended for treatment of AD and other NDs. Thus, only four low-molecular agents are extensively used in the therapy of AD (donepezil, rivastigmine, galantamine, memantine) and one monoclonal antibody agent—aducanumab, which was approved in 2021. The former three drugs are cholinesterase inhibitors, whereas memantine is an NMDA-receptor channel blocker. It should be noted that all these drugs only provide some relief to the symptoms of AD. The fact that only a small number of drugs has been approved for the pharmacotherapy of AD and that these agents provide only partial amelioration of symptoms without altering the progression of the disease attests to the multifactorial nature of the disease and our incomplete understanding of its pathogenesis [4,5]. Therefore, the construction of multifunctional disease-modifying agents that can act simultaneously on several targets thought to be involved in the pathogenesis of the disease is a current trend in the design of new effective treatments of these pathologies [6,7].

**Figure 1:**
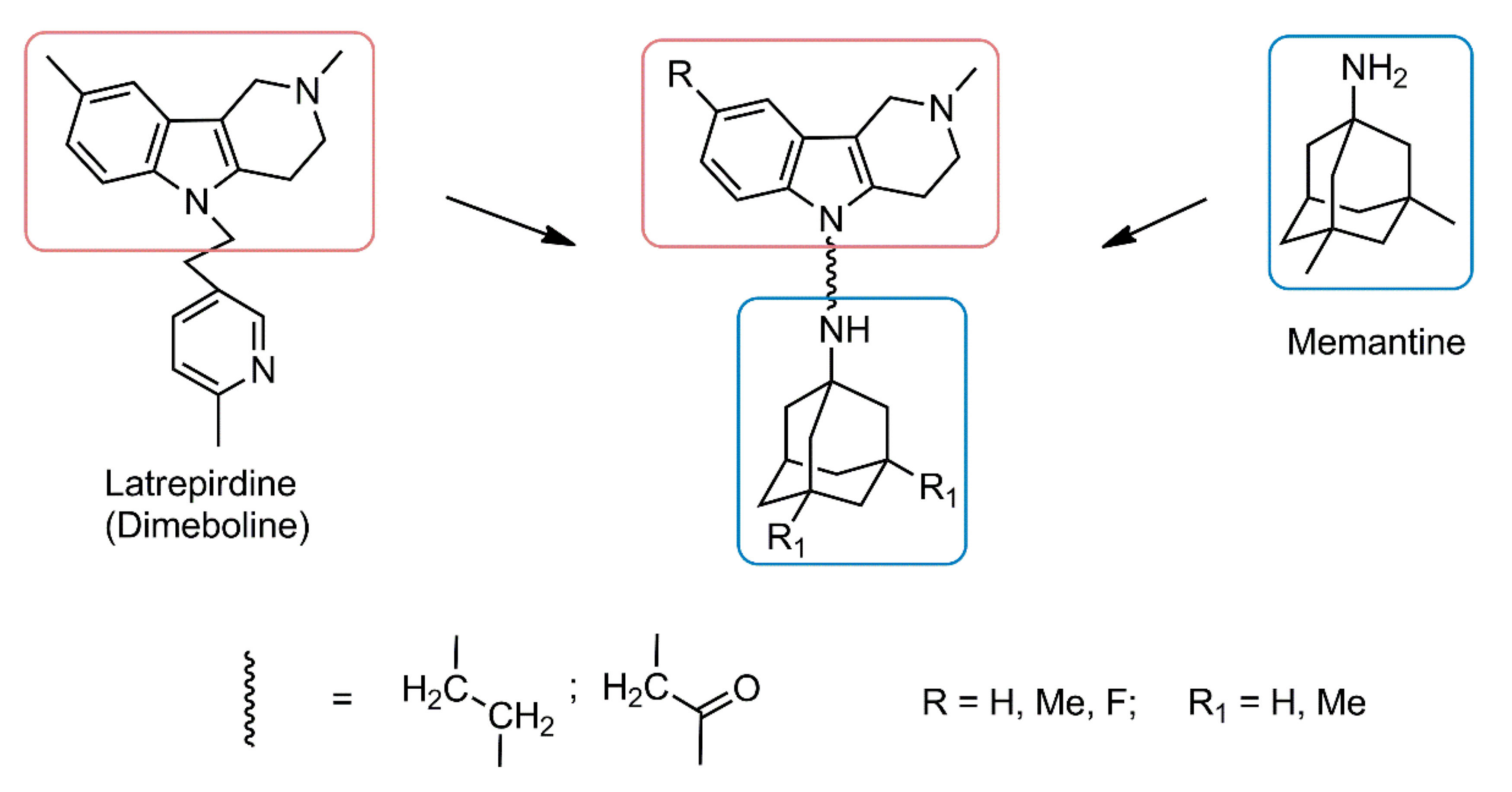
Design of aminoadamantane–γ-carboline conjugates.

Previously, we used approaches to the construction of several types of multifunctional ligands including those based on carbazole–γ-carboline, carbazole–aminoadamantane and γ-carboline–phenothiazine conjugates [8,9,10,11,12,13]. In the current work, we investigated approaches to the design of new binary compounds based on aminoadamantane and γ-carboline and characterized the synthesized compounds. These compounds can be considered as conjugates of the drug memantine, acting as a blocker of brain NMDA receptors [14,15], and the drug dimebon. The latter showed strong neuroprotective properties in phase II clinical trials [16]; however, because of an abnormal placebo effect in phase III clinical trials, it was not further developed [17]. Meanwhile, subsequent studies of dimebon and its analogs provided convincing evidence that this compound has a significant mitoprotective effect, inhibits the development of proteinopathy and affects a number of enzyme and receptor systems (such as acetylcholinesterase (AChE) and butyrylcholinesterase (BChE), 5-HT7 and 5-HT6 subtype serotonin receptors, NMDA- and AMPA-subtype glutamate receptors and some others involved in neurotransmission) [18,19,20,21,22]. Therefore, a combination of active pharmacophores (aminoadamantane and γ-carboline in one chemical structure is of great interest. Our design of the conjugates is presented in Figure 1.

We studied the interactions of the synthesized conjugates with the two binding sites of the NMDA receptor: the intrachannel (MK-801-binding) and peripheral (ifenprodil-binding) sites. We used enzyme kinetics and molecular docking to investigate interactions of the conjugates with enzymes of the cholinergic nervous system, acetylcholinesterase (EC 3.1.1.7, AChE) and butyrylcholinesterase (EC 3.1.1.8, BChE), and the structurally related enzyme carboxylesterase (EC 3.1.1.1, CES), the inhibition of which can lead to unwanted drug-drug interactions (anti-target). We also evaluated the ability of the new conjugates to bind to the peripheral anionic site (PAS) of AChE from *Electrophorus electricus* (*Ee*AChE) and competitively displace its selective ligand propidium iodide as an indicator of their potential ability to prevent AChE-induced β-amyloid aggregation. In order to evaluate the potential toxicity and the neuroprotective effect of the compounds, we evaluated the action of the new aminoadamantane–γ-carboline conjugates on the mitochondrial potential and calcium-induced mitochondrial permeability transition (MPT) pore opening, respectively. The possibility to compensate microtubule destabilization upon tauopathy was assessed based on the ability of the new compounds to stimulate the polymerization of tubulin and microtubule-associated proteins.

## 2. Results and Discussion

### 2.1. Chemistry

**Scheme 1:**
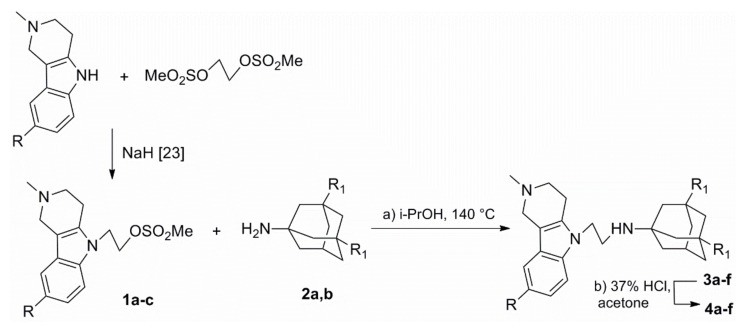
Synthesis of compounds **4a**–**f**. Reagents and conditions: (**a**) i-PrOH, 140 °C, sealed tube, 3 days, (**b**) 37% (*v*/*v*) HCl, acetone.

**Scheme 2:**
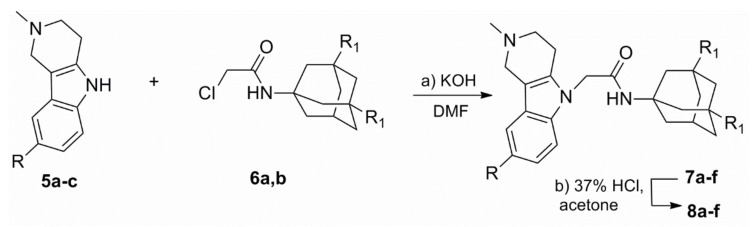
Synthesis of compounds **8a**–**f**. Reagents and conditions: (**a**) KOH, 20 °C, 3 h, (**b**) 37% (*v*/*v*) HCl, acetone.

The target compounds were synthesized as outlined in Scheme 1 and Scheme 2. A series of aminoadamantane–γ-carboline conjugates linked via an ethylene spacer was synthesized starting from methanesulfonates **1a**–**c**. Methanesulfonates **1a**–**c** were prepared by the reactions of tetrahydro-γ-carbolines with ethane-1,2-diyl dimethanesulfonate in the presence of an equimolar amount of sodium hydride as described [23] and were then heated with 1-aminoadamantanes **2a**,**b** in sealed tubes for 3 days at 140 °C. Conjugates **3a**–**f** were isolated by column chromatography and converted into corresponding hydrochlorides **4a**–**f**.

A series of aminoadamantane–γ-carboline conjugates linked via an oxoethylene spacer was synthesized by the reaction of chloroacetylaminoadamantanes with γ-carbolines. γ-Carbolines **1a**–**c** were alkylated with *N*-(adamantan-1-yl)chloroacetamides **6a,b** in the presence of KOH to give conjugates **7a**–**f** in high yields, and the latter compounds were transformed into corresponding hydrochlorides **8a**–**f**. All the synthesized compounds were characterized by ^1^H, ^13^C and ^19^F NMR spectroscopy and elemental analysis.

The IR spectra showed the characteristic appearance of an absorption band in the 3450–3400 (NH), 2910–2900 (CH_2_), 2854–2840 (NCH_3_), 1679–1675 (C=O), 1545–1542 (NH of amide) and 1464–1452 (CH of CH_2_) cm^−1^ range. ^1^H and ^13^C NMR spectra represent a superposition of the signals of the tetrahydro-γ-carboline ring, aminoadamantane fragment and ethylene or oxoethylene spacers. Characteristic signals of two spacers in ^1^H NMR spectra appear in the ranges of 4.3–4.7 ppm for the NCH_2_CH_2_NH spacer of compounds **4a**–**f** and in the ranges of 4.68–4.76 ppm for the NCH_2_C(O) spacer of compounds **8a**–**f**. Signals of ^13^C NMR spectra in the ranges of 56–58 ppm were assigned to methylene of the NCH_2_CH_2_NH spacer of compounds **4a**–**f**, while the NCH_2_C(O) spacer of compounds **8a**–**f** demonstrate signals of methylene protons at 46 ppm and 166 ppm of the carbonyl group.

### 2.2. Effect of Aminoadamantane–γ-Carboline Conjugates on NMDA Receptors

The glutamatergic system of the central nervous system (CNS) is known to play a key role in memory consolidation and neurodegenerative processes leading to impairment of cognitive functions and memory during such diseases as AD and senile dementia [24]. Ionotropic glutamate receptors coupled to ion channels, in particular NMDA-subtype glutamate receptors, play an important role in these processes [25,26,27]. Compounds that are able to block NMDA receptors have long been considered as potential treatments for neurodegenerative diseases [28,29,30]. The drug memantine, the best-known NMDA receptor blocker, is used in clinical practice to improve memory and cognitive functions in patients with later stages of AD and some other diseases [14,31]. It is assumed that memantine interacts mainly with the binding site within the NMDA receptor ion channel [32]. Specific (allosteric) binding sites on NR2B subunits of the NMDA receptor are considered as another potential target in the search for neuroprotective and cognitive-enhancing agents. The NR2B subunit is involved in neurodegenerative processes in patients with Parkinson’s and Huntington’s diseases as well as AD [33]. Therefore, efficient modulators of GluN2B-containing NMDARs are a promising tool for further investigation and potential treatment of these diseases [34,35,36].

The effect of the synthesized compounds on the two binding sites of the NMDA receptor, namely, the intrachannel (MK-801-binding) and peripheral (ifenprodil-binding) sites, was studied by a radiolabeled ligand displacement assay using [^3^H]-MK-801 and [^3^H]-ifenprodil as radioactive-labeled specific ligands, respectively). The results are summarized in Table 1. 

Based on the results in Table 1, the following conclusions can be made about the effect of the spacer structure and the pharmacophore scaffolds on anti-NMDA activity:

In all cases, the use of the 1-oxoethylene spacer instead of the ethylene one substantially (by 1–1.5 orders of magnitude) impairs the binding of the compounds to both sites of the NMDA receptor. 

Compounds bearing the ethylene spacer (**4a**–**4f**) have similar *K*_i_ values in the micromolar range almost regardless of the nature of the substituents in the polycyclic moieties (conjugated scaffolds). The *K*_i_ values toward the MK-801-binding site for these compounds are close to that for memantine, for which this binding site of the NMDA receptor is the main target of action. In particular, monomethyl-substituted 1,2,3,4-tetrahydro-γ-carbolines (compounds **4a** and **4d**) are the most active compounds in this respect. 

Unexpectedly, the compounds bearing the ethylene spacer (**4a**–**4f**) show high (micromolar) activity toward the ifenprodil-binding site of the NMDA receptor, as opposed to memantine and dimebon, which, being structural analogs of conjugated polycyclic scaffolds, do not exhibit specific activity toward this site of the NMDA receptor. 

### 2.3. Esterase Profiles of Aminoadamantane–γ-Carboline Conjugates and Their Ability to Displace Propidium Iodide from the AChE PAS

Cholinergic deficit is a characteristic feature of AD. It is associated with the selective loss of cholinergic neurons in the nucleus basalis of Meynert in early stages of the disease and is largely responsible for the impairment of cognitive functions [37,38]. Inhibitors of AChE suppress its classical function of hydrolysis of the neurotransmitter acetylcholine, resulting in an increase in the activation of cholinergic neurons and stimulation of cognitive functions [37,39]. 

The inhibition of another cholinesterase, BChE, also facilitates the improvement of cognitive functions. It is worth noting that BChE inhibition is particularly effective in the case of disease progression, when AChE activity decreases, while BChE activity gradually increases. Hence, BChE is of great interest as a therapeutic target for decreasing the cholinergic deficit [40,41] in advanced stages of the disease. It should be emphasized that selective BChE inhibitors increase the acetylcholine level in the brain, having no classical side effects typical of AChE inhibitors [42,43]. Therefore, considerable recent attention has been focused on the search for selective BChE inhibitors, as well as compounds inhibiting both cholinesterases, AChE and BChE, because in this case the effective therapeutic action would be expected to be retained both in early and later stages of AD progression [42,44,45]. 

In the current study, the evaluation of the esterase profile of the new compounds included also the determination of inhibitory activity against carboxylesterase (CES), which is structurally similar to cholinesterases. This enzyme catalyzes the hydrolysis of many drugs containing ester groups [46,47] and is considered as an anti-target, because the ability of anticholinesterase agents, used in the treatment of AD, to inhibit CES can lead to unwanted drug-drug interactions.

Another important aspect of the action of cholinesterase inhibitors is associated with the aggregation and deposition of β-amyloid peptides in the brain, which plays a key role in the appearance and progression of AD [48,49]. It was shown that, apart from the classical function of acetylcholine hydrolysis, AChE exhibits β-amyloid proaggregation activity [50] due to the ability of the PAS of the enzyme to interact with soluble β-amyloid peptides, thereby promoting their aggregation [9,51,52,53,54]. Hence, AChE inhibitors that interact with the AChE PAS can be considered as potential antiaggregation agents [53,55,56,57]. It is assumed that BChE is also involved in the formation and/or maturation of β-amyloid plaques, thereby contributing to the pathogenesis of AD [58,59,60,61]. Therefore, AChE inhibitors and inhibitors of both cholinesterases are of interest in anti-amyloid strategies. 

#### 2.3.1. Evaluation of the Esterase Profile of Aminoadamantane–γ-Carboline Conjugates

To evaluate the esterase profiles of aminoadamantane–γ-carboline conjugates, their inhibitory activity against three structurally similar serine esterases, AChE, BChE and CES was determined. We used human erythrocyte AChE, equine serum BChE and porcine liver CES, which were shown to be suitable for this purpose in our previous studies [11,12,47,62,63]. The results showed (Table 2) that all of the conjugates were more potent inhibitors of BChE compared to AChE and that they had very weak inhibitory activity against CES. The conjugates containing the memantine fragment were somewhat more effective than their amantadine analogs as BChE inhibitors. 

The spacer structure had an appreciable effect on the inhibitory activity of the conjugates. Conjugates **4** linked via the ethylene spacer were effective and selective BChE inhibitors. The replacement of the ethylene spacer by the 1-oxoethylene one (conjugates **8**) led to a decrease in BChE inhibition (see Table 2, evidenced by the comparison of **4a**–**c** and **8a**–**c, 4d**–**f** and **8d**–**f**). Regarding the effect of the structure of the γ-carboline fragment, the 2-Me and 2-Me, 8-F derivatives exhibited the highest activity in all four groups. Conjugates **4a** and **4c** that inhibited BChE in the submicromolar concentration range were the most effective.

Whereas the synthesized conjugates were more effective against BChE than AChE, the spacer structure also had an appreciable effect on the inhibitory activity of the conjugates against AChE. Conjugates **4** linked via an ethylene spacer were modest AChE inhibitors. However, their analogs **8** linked via the oxoethylene spacer were substantially less effective, and at a concentration of 20 µM, the degree of inhibition varied from 5 to 22% (see Table 2). 

#### 2.3.2. Kinetic Studies of AChE and BChE Inhibition

**Figure 2:**
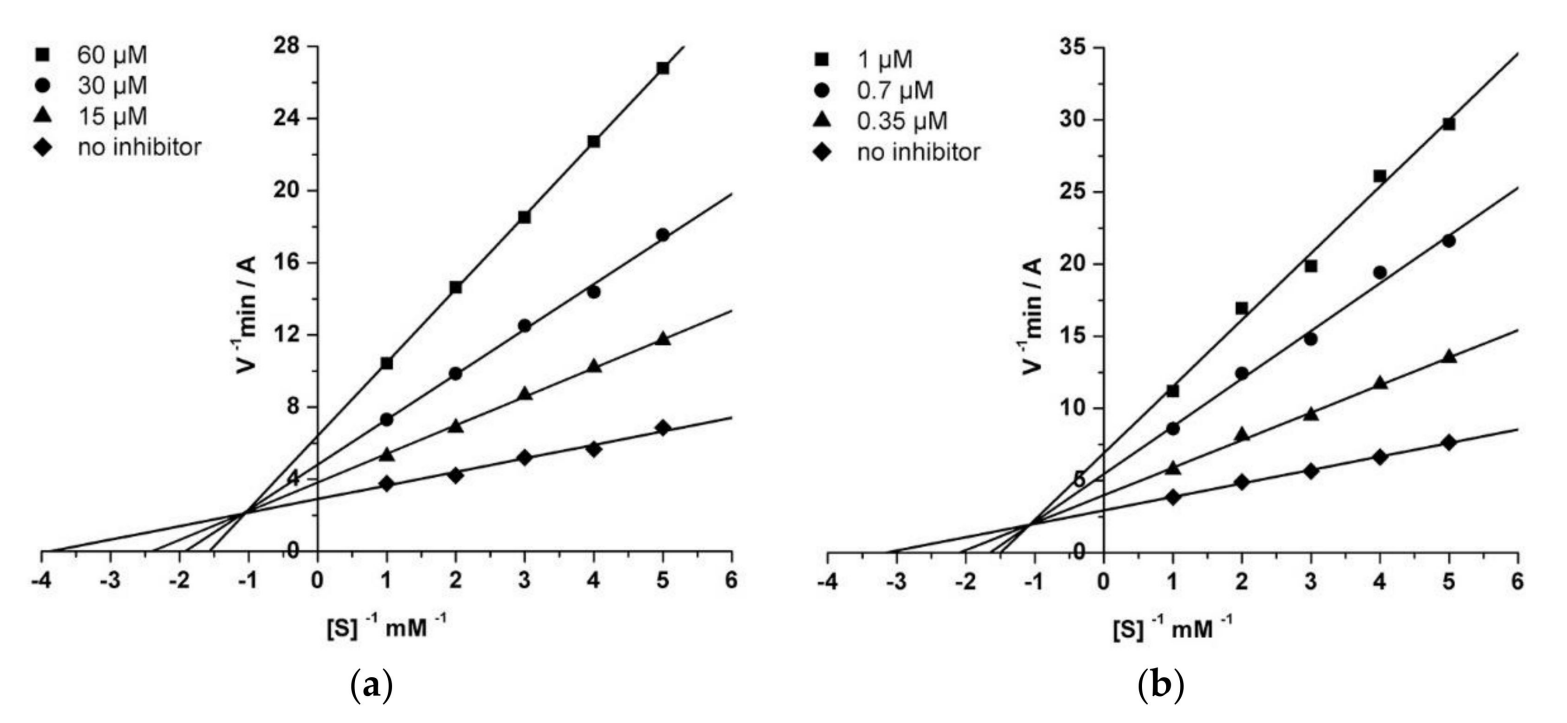
Kinetics of the steady-state inhibition of AChE (**a**) and BChE (**b**) by compound **4c**. The data are presented as Lineweaver–Burk double-reciprocal plots.

Studies of the kinetics and mechanism of AChE and BChE inhibition by the synthesized aminoadamantane–γ-carboline conjugates were conducted using compound **4c**. The graphical analysis of the kinetic data on AChE (Figure 2a) and BChE (Figure 2b) inhibition by compound **4c** by Lineweaver–Burk double-reciprocal plots showed changes in both *K*_m_ and *V*_max_, which is indicative of a mixed type of inhibition. Inhibition constants for AChE were 13.70 ± 0.02 μM (*K*_i_, the competitive component) and 45.5 ± 3.8 μM (α*K*_i_, the non-competitive component). Corresponding values for BChE were 0.23 ± 0.02 µM (*K*_i_) and 0.70 ± 0.01 µM (α*K*_i_).

#### 2.3.3. Molecular Docking to BChE

The molecular docking of the conjugates to BChE accounts for some of the observed structure–activity relationships. Even though binding energies could not be directly related to IC_50_ values [64], the rank order of docking scores for the most potent 4 compounds and the least potent compound against BChE were in agreement with the rankings for experimentally determined potencies. (Table S1). Moreover, the docking scores and inhibitor potencies for all compounds were well correlated after converting IC_50_ values to pIC_50_ values: r = 0.725; *p* = 0.003; *n* = 14 (r—Pearson correlation coefficient, which can be used because in this case, both the pIC_50_ and the docking scores were normally distributed). 

**Figure 3:**
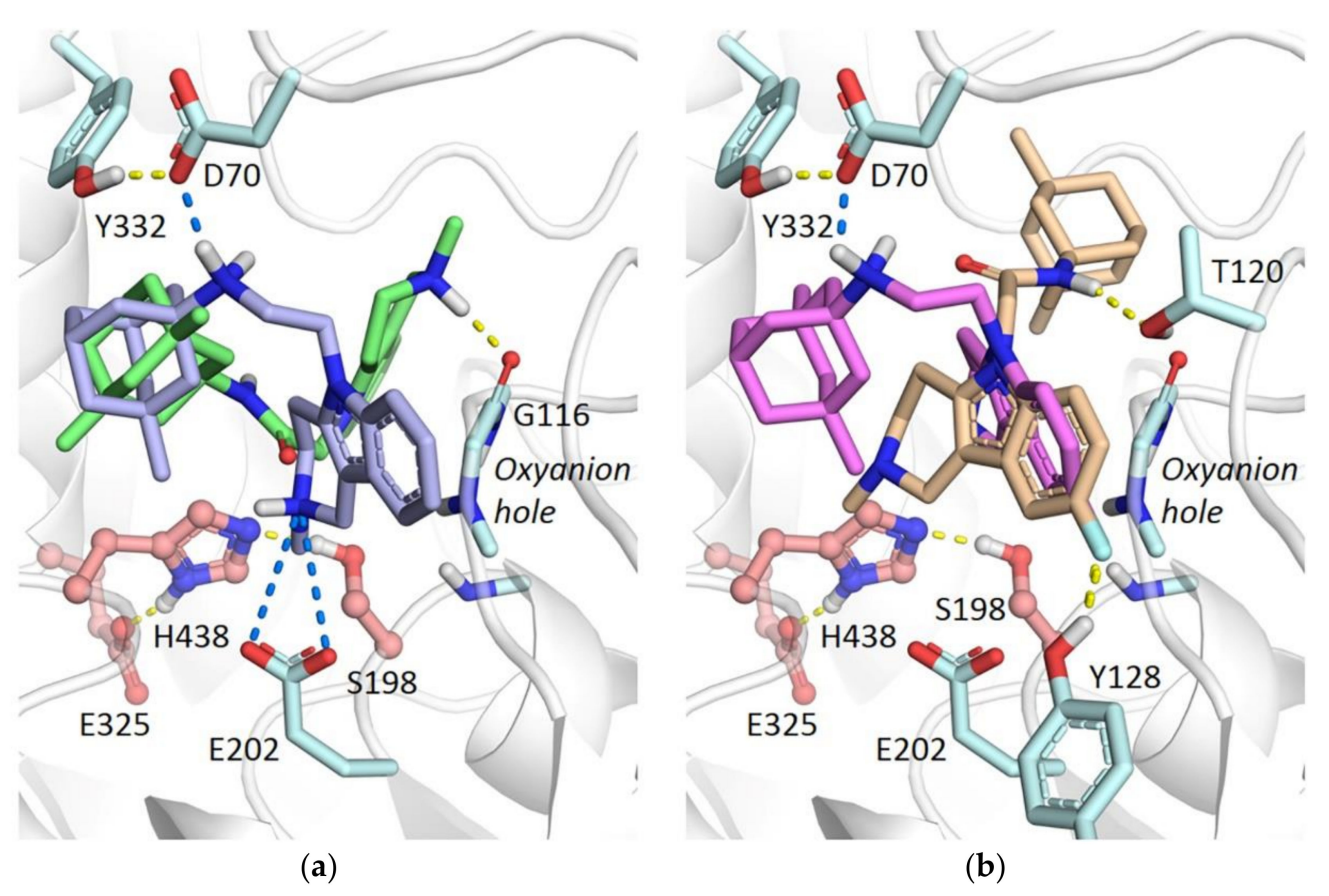
Docking of conjugates linked via ethylene and 1-oxoethylene spacers to BChE: (**a**), comparison of **4a**, the ethylene spacer (carbon atoms are shown in light blue) and **8a** the 1-oxoethylene spacer (light green); (**b**), comparison of **4c**, the ethylene spacer (purple) and **8c**, the 1-oxoethylene spacer (sandy colored).

It was found that memantine and amantadine fragments were bound within the active site gorge of BChE so that the protonated aminoadamantane group of conjugates **4** linked via the ethylene spacer formed an ion pair with Asp70 (Figure 3a). In contrast, the relevant group in conjugates **8** linked via the 1-oxoethylene spacer is an amide; consequently, it is non-charged, resulting in a decrease in the number of specific interactions, thus explaining the observed reduction of inhibitory activity (see Figure 3a). The hydrophobic pocket within the active site gorge of BChE was the best-docked pose of the amantadine/memantine group, as discussed previously for memantine–carbazole conjugates [9]. The presence of the amantadine/memantine group alters the position of the γ-carboline fragment of conjugates compared to the binding pose of their prototype dimebon (Figure S1a). However, the binding pose of compound **8a** with the 1-oxoethylene spacer was very close to that of the reference compound donepezil (Figure S1b).

For the conjugates linked via the 1-oxoethylene spacer, another, although somewhat less stable, binding pose is also possible. In this pose, the memantine group points toward the entrance of the active site gorge, thereby providing hydrogen bonding between the amide group and Thr120 (see Figure 3b). 

**Figure 4:**
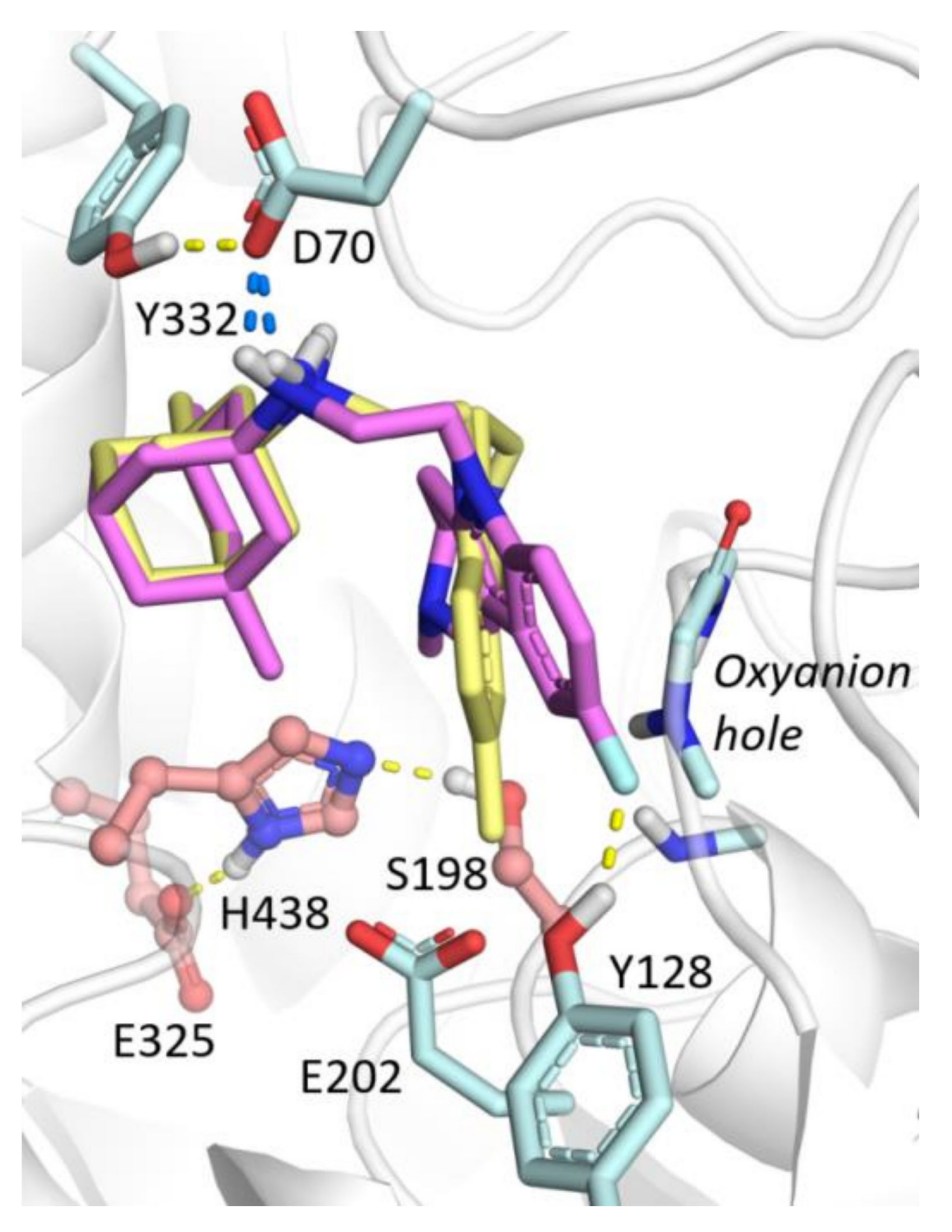
Docking of memantine derivatives, which differ by the structure of the γ-carboline fragment, to BChE: 2-Me, 8-F derivative **4c** (carbon atoms are shown in purple) and its dimethyl analog **4b** (carbon atoms are shown in yellow).

A comparative docking to the active site of BChE of compounds **4b** and **4c**, which differ by the structure of the γ-carboline fragment, demonstrated that the fluorine atom could act as an acceptor of a hydrogen bond with Tyr128. This interaction improves the binding of conjugate **4c** [65] compared to the dimethyl derivative **4b** and is responsible for its higher inhibitory activity (Figure 4).

#### 2.3.4. Molecular Docking to AChE

**Figure 5:**
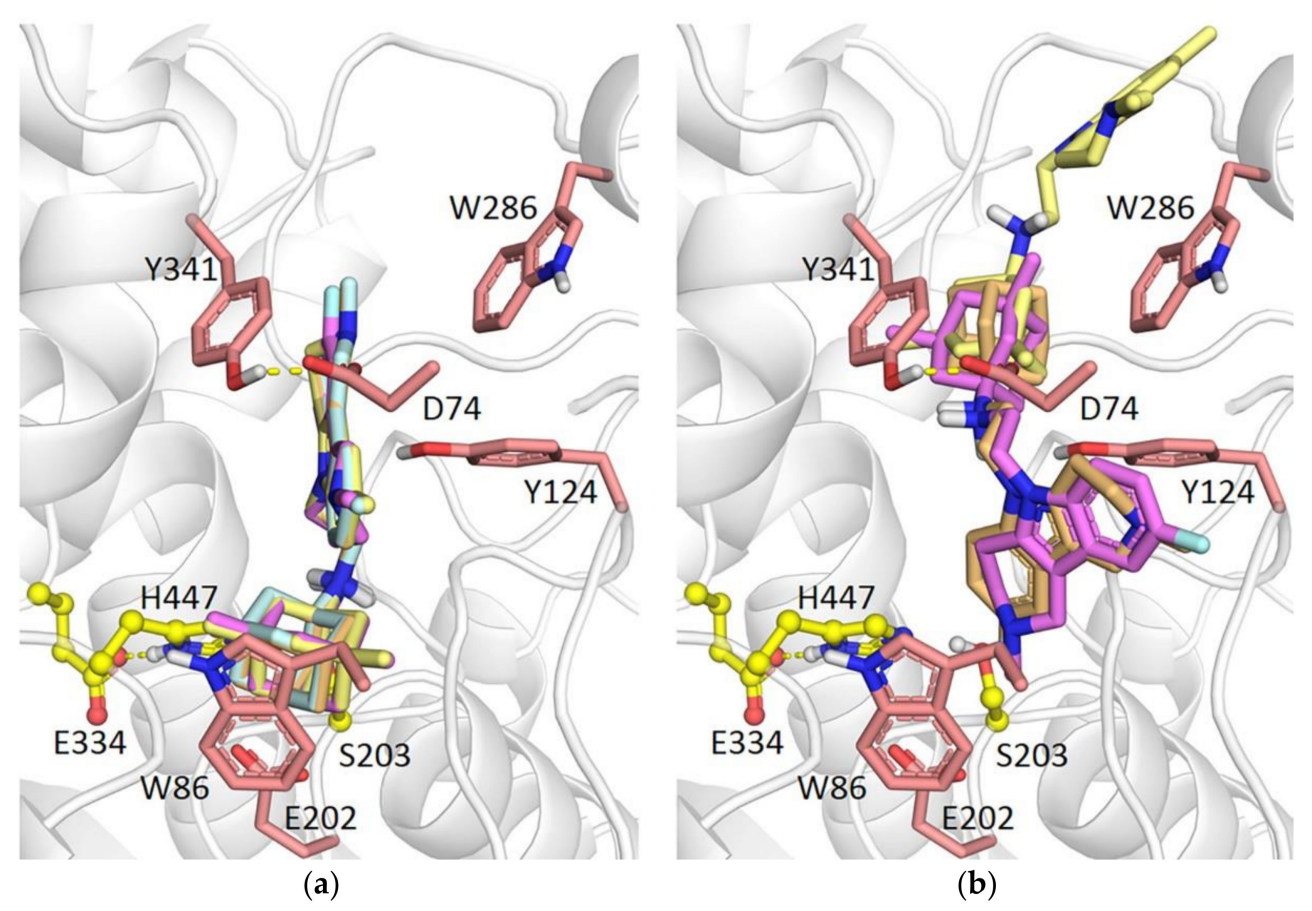
Docking of memantine and amantadine derivatives linked via the ethylene spacer to AChE (carbon atoms are shown in yellow for **4b**, blue for **4e**, purple for **4c** and orange for **4f**). Panel (**a**): the best energy-scored pose based on molecular docking, which could be kinetically hindered. Panel (**b**): the binding pose ranked second in energy, with the amantadine/memantine group bound to the PAS. The γ-carboline group can bind to both the CAS and PAS.

The molecular docking to AChE was performed for conjugates **4** with memantine (**4b**,**c**) and amantadine (**4e**,**f**) fragments linked via the ethylene spacer. The best binding pose was obtained with the amantadine/memantine group bound in the catalytic active site (CAS) of AChE and the γ-carboline group partially occupying the PAS (Figure 5a). Here, the amantadine/memantine moiety takes the place of the phenyl group of donepezil, the reference compound (Figure S2a). The docked position of donepezil was identical to the binding pose obtained with X-ray crystallography [66,67]. A clear difference in π-π interactions with Trp86 and Trp286 could contribute to the lower binding affinities of compounds **4** compared to donepezil. Moreover, taking into account that the CAS of AChE is separated from the PAS by the so-called bottleneck, the penetration of the bulky amantadine/memantine group (much bigger than phenyl) into the CAS could be kinetically hindered [68]. Consequently, the binding pose ranked second in energy, with the amantadine/memantine group in the PAS (Figure 5b), seemed more reasonable and should ensure efficient displacement of the selective PAS ligand, propidium iodide. Here it also overlapped with the position of donepezil (Figure S2b). In this case, the γ-carboline group could bind to both the PAS and CAS, and these poses were characterized by similar binding energies. Compared with the docked pose of dimebon, the positions of the γ-carboline fragments of conjugates **4** in the CAS were identical (Figure S2b). Overall, these docking results were in good agreement with the results of the kinetic study of inhibition, which showed a mixed type of inhibition.

#### 2.3.5. Evaluation of Conjugates Ability to Displace Propidium Iodide from the EeAChE PAS

Taking into account the β-amyloid proaggregation properties of AChE [50], we evaluated conjugates **4** and **8** for their ability to displace the selective PAS ligand, propidium iodide, from the *Ee*AChE PAS. This method is widely used as a screening approach to assess the potential ability of compounds to block AChE-induced β-amyloid aggregation, because when the compounds bind to the AChE PAS, they prevent the binding of β-amyloid, thereby inhibiting its aggregation [53,55,69,70,71,72,73]. The method is described in detail in [74]. *Ee*AChE was used owing to its high degree of purification, high activity, lower cost than human AChE and essential congruence of the two enzyme structures [74]. Donepezil, a mixed-type AChE inhibitor, for which the ability to block AChE-PAS-induced Aβ aggregation has been demonstrated [53], was used as a reference compound. The results are presented in Table 2.

Conjugates **4** linked via the ethylene spacer exhibited activities equal to or higher than that of donepezil. They displaced propidium iodide from the *Ee*AChE PAS by 10–18%. Meanwhile, conjugates **8** linked via the 1-oxoethylene spacer very poorly inhibited the catalytic activity of AChE and, in general, could not effectively displace propidium. 

Therefore, the data on the displacement of propidium iodide from the AChE PAS and the results of kinetic studies and molecular docking show that conjugates **4** linked via the ethylene spacer can bind to both the CAS and the PAS of AChE. Due to binding to the AChE PAS, compounds **4**, apart from effective BChE inhibition and moderate AChE inhibition, could potentially block AChE-induced β-amyloid aggregation and thereby exhibit a disease-modifying effect.

### 2.4. Effect of Aminoadamantane–γ-Carboline Conjugates on the Functional Properties of Isolated Rat Liver Mitochondria 

In the drug design for the treatment of NDs, an important issue is to provide neuroprotective effects against processes causing cell death. A promising target for such activity is the MPT, a key step in the cell death cascade implicated in neurodegeneration [75,76]. According to some authors, the neuroprotective action of dimebon, a prototype of the γ-carboline pharmacophore contained in our conjugates, could be ascribed to its ability to increase the resistance of mitochondria to the induction of the MPT [18,19,20]. Consistent with this view, our primary panel of mitochondrial tests showed promise in the search for new drugs for the treatment of NDs [77,78], and these results were echoed in our studies of bipharmacophoric compounds [8,9,12] and related indole–aminoadamantane conjugates [9].

To assess the interactions of the new conjugates with mitochondria, we evaluated their effects on mitochondrial membrane potential in the presence of substrates of complex I (glutamate and malate) and complex II (succinate) of the mitochondrial respiratory chain. None of the tested compounds affected the membrane potential of isolated mitochondria; moreover, all of them reduced spontaneous depolarization. Furthermore, the conjugates decreased calcium-induced mitochondrial depolarization. These results indicated that these compounds lacked mitochondrial toxicity and allowed us to suggest the possibility of a protective effect, such as prevention of the induction of the MPT. 

Therefore, in the next step, we evaluated the effect of the tested compounds on Ca^2+^-induced mitochondrial swelling, a reflection of the opening of the MPT pore. Inhibition of MPT pore opening would be an important indicator of potential neuroprotective activity. The results are summarized in Table 3. All conjugates exhibited MPT-inhibitory activity. Under these conditions, the efficacy of a number of the conjugates was similar to or even somewhat higher than that of dimebon. The conjugates linked via the ethylene spacer (**4a**–**f**) more effectively decreased the maximum rate of mitochondrial swelling at a concentration of 30 µM (by more than 70% relative to the reference compound, which is comparable with the activity of dimebon). However, the introduction of the 1-oxoethylene spacer reduced the ability to suppress the MPT.

**Figure 6:**
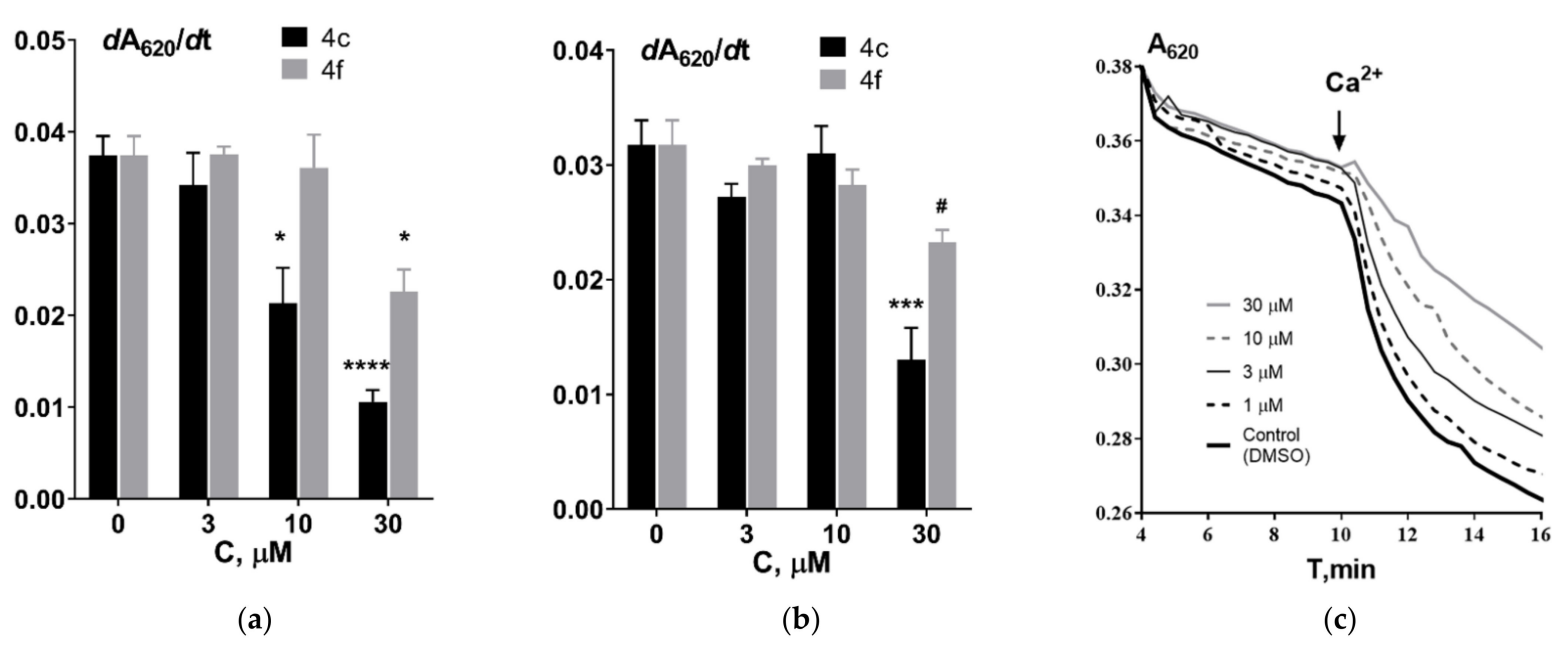
Effect of compounds **4c** and **4f** on the rate of Ca^2+^-induced mitochondrial swelling. (**a**) in the presence of the substrate of complex II and the inhibitor rotenone of complex I. (**b**) compared to mitochondria energized by glutamate and malate. (**c**) representative curve of **4c** influence on the kinetics of mitochondrial swelling in the presence of the substrate of complex II and the inhibitor rotenone of complex I. The Ca^2+^ concentration was 12.5 µM. The significance of the differences was estimated by two-factor dispersion analysis ANOVA with Tukey’s post-hoc multiple comparison test (* *p* ≤ 0.05, *** *p* ≤ 0.001, **** *p* ≤ 0.0001, comparison with the control group) and Student’s *t*-test (# *p* ≤ 0.05, comparison with the control group).

The concentration dependences of the inhibition of Ca^2+^-induced swelling (Figure 6) of mitochondria in the presence of different substrates of the mitochondrial respiratory chain were determined for a pair of active compounds **4c** and **4f,** which differed in the type of aminoadamantyl substituent. The results were indicative of the inhibitory effect on the Ca^2+^-induced mitochondrial pore opening. The conjugates more effectively inhibited Ca^2+^-dependent MPT pore opening in the presence of the substrate of complex II and the inhibitor rotenone of complex I (Figure 6a) compared to mitochondria energized by glutamate and malate (Figure 6b). It is worth noting that compound **4c** containing the dimethylaminoadamantyl (memantine) substituent had stronger mitoprotective properties than **4f** with aminoadamantyl fragment.

After the addition of the tested compounds to a suspension of mitochondria, the spontaneous depolarization was significantly inhibited compared to the control group (data not shown). There are many possible mechanisms of this effect; the most probable being reduction of spontaneous MPT pore opening, suppression of free radical production by mitochondria or inhibition of membrane lipid peroxidation [79]. 

It should also be noted that the conjugates showed no cytotoxic properties in the evaluation of the effect of 30 µM of tested compounds on the viability of SH-SY5Y neuroblastoma cells by the MTT assay: the percentage of mitochondrial dehydrogenase activity in the test cells with the conjugates did not differ from that in the control (data not shown).

### 2.5. Effect of Aminoadamantane–γ-Carboline Conjugates on Tubulin Polymerization

Previously, we found compounds in the series of indole–aminoadamantane conjugates, namely tetrahydrocarbazole–aminoadamantane conjugates, which could significantly stimulate tubulin polymerization to form microtubules with normal structure [9]. The stabilization of microtubules as a compensation of the impaired function of the microtubule-associated τ-protein, which is caused by its hyperphosphorylation and aggregation, is a highly promising disease-modifying therapy for tauopathies as NDs [80]. 

**Figure 7:**
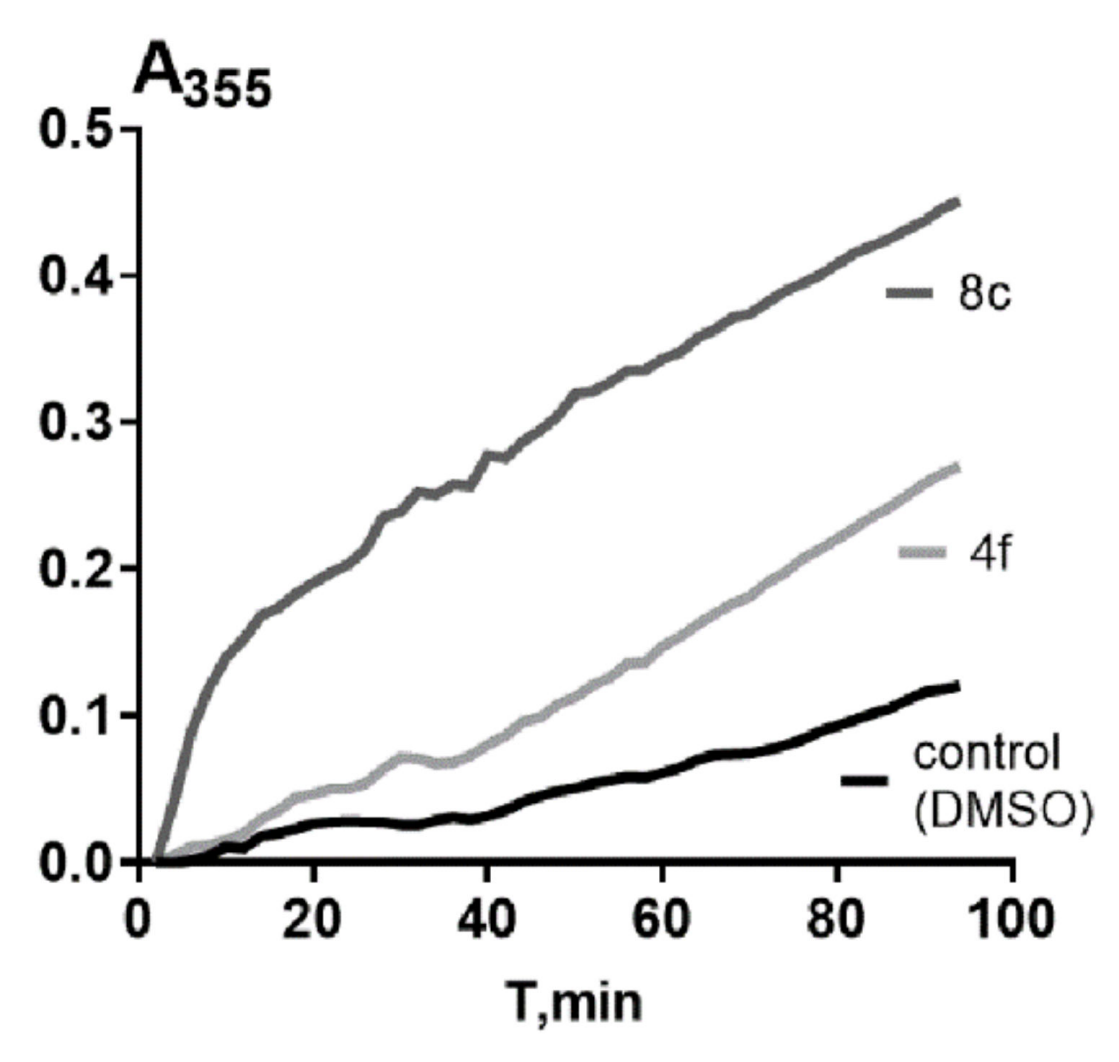
Representative curves of effect of compounds **8c** and **4f** on GTP-dependent Tb+MAP polymerization.

Therefore, we performed primary screening of this series of aminoadamantane–γ-carboline conjugates for their effect on the polymerization of a crude preparation of tubulin and microtubule-associated proteins (Tb+MAP). An increase in the rate of Tb+MAP polymerization was observed for all the conjugates under study. For the most active fluorine-containing conjugates **4f** and **8c** linked via the ethylene and oxoethylene spacer, respectively, the rate of Tb+MAP polymerization increased by more than a factor of 3.5 (see Table 3 and Figure 7). In the series of the conjugates linked via the oxoethylene spacer, the conjugates containing the dimethylaminoadamantyl (memantine) moiety caused the most substantial increase in the polymerization rate. In contrast, in the series of conjugates linked via the ethylene spacer, the compounds containing the unsubstituted adamantyl group were found to be the most active. The replacement of the methyl substituent at the 8-position of the γ-carboline ring with fluorine did not lead to an appreciable change in the activity of the compounds.

## 3. Materials and Methods

### 3.1. Synthesis Method and Spectral Data

FT IR spectra were obtained on a Bruker IFS-113v spectrometer (Bruker Analytik GmbH, Silberstreifen, Germany) and recorded as KBr pallets with absorptions reported as cm^−1^. The ^1^H, ^13^C and ^19^F NMR spectra were recorded on a Bruker DXP spectrometer (Bruker, Billerica, MA, USA) at 200, 50 and 188 MHz, respectively, in DMSO-d_6_ using tetramethylsilane as an internal standard and CCl_3_F as an external standard. Chemical shifts are reported in ppm on the *δ* scale. Coupling constants are given in Hertz (Hz). Melting points were measured in open capillary tubes with a STUART SMP20 melting point apparatus (Cole-Parmer, Vernon Hills, IL, USA) and uncorrected. The elemental analysis was carried out on an Elementar vario MICRO cube CHNS/O analyzer. Chromatography was performed on Alfa Aesar 220–440 mesh silica gel. The synthesis of compounds **1a**–**c** was described in [23]. Gamma-carbolines **5a**–**c** were prepared as described in [81]; chloroacetylamides **6a**,**b**, in [82]. 

#### 3.1.1. General Procedure for the Preparation of Compounds **4a**–**f**

Solutions of compound **1a**–**c** (1 mmol) and 1-aminoadamantane **2** (1 mmol) in i-PrOH (10 mL) were placed in sealed tubes and heated for 3 days at 140 °C. The solvent was evaporated, and the residue was purified by column chromatography on silica gel (60 mesh, methanol—chloroform, 1:10, as the eluent). To a solution of compound **3** (0.5 mmol) in acetone (5 mL), 37% (*v*/*v*) HCl (0.1 mL) was added, and the precipitate that formed was filtered off. 

3,5-Dimethyl-*N*-(2-(2-methyl-3,4-dihydro-1*H*-pyrido[4,3-*b*]indol-5(2*H*)-yl)ethyl)-adamantane-1-amine dihydrochloride (**4a**). Brown solid. Yield 35%. Mp 206–207 °C. FT IR (KBr ν_max_ in cm^−1^): 3432 (NH), 2914 (CH_2_), 2854 (NCH_3_), 1618 (C=C), 1454 (CH of CH_2_). ^1^H NMR (200 MHz, DMSO-d_6_): δ = 0.85 (s, 6H, C_Ad_H_3_), 1.00–1.22 (m, 2H, C_Ad_H_2_), 1.22–1.44 (m, 4H, C_Ad_H_2_), 1.46–1.84 (m, 6H, C_Ad_H_2_), 2.16 (m, 1H, C_Ad_H), 3.14 (s, 3H, CH_3_N), 3.38–3.70 (m,4H, CH_2_), 3.81–4.12 (m, 1H, CH_2_), 4.20–4.42 (m, 1H, CH_2_), 4.94–5.20 (m, 1H, CH_2_), 5.35–5.70 (m, 3H, CH_2_), 7.04–7.23 (m, 2H, CH_Ar_), 7.46 (d, 1H, C_Ar_H, ^3^*J*_HH_ = 7.7 Hz), 7.69 (d, 1H, C_Ar_H, ^3^*J*_HH_ = 7.7 Hz), 10.65 (br. S, 2H, NH), 13.80 (br. S, 1H, NH^+^CH_3_). 19.25 (CH_2_), 29.43 (CH), 29.96 (CH_3_), 31.90 (C), 36.06 (CH_2_), 37.72 (CH_2_), 41.42 (CH_2_), 41.52 (CH_2_), 43.24 (CH_3_), 49.08 (CH_2_), 49.46 (CH_2_), 49.90 (CH_2_), 57.94 (C), 101.64 (C), 109.33 (CH), 117.28 (CH), 119.23 (CH), 121.37 (CH), 124.15 (C), 130.56 (C), 135.61 (C). Anal. C 67.42, H 8.66, N 9.21%. Calcd. For C_26_H_39_Cl_2_N_3_, C 67.23, H 8.46, N 9.05%.

*N*-(2-(2,8-Dimethyl-3,4-dihydro-1*H*-pyrido[4,3-*b*]indol-5(2*H*)-yl)ethyl)-3,5-dimethyladamantane-1-amine dihydrochloride (**4b**). Brown solid. Yield 34%. Mp 205–206 °C. FT IR (KBr ν_max_ in cm^−1^): 3432 (NH), 2906 (CH_2_), 2842 (NCH_3_), 1622 (C=C), 1457 (CH of CH_2_). ^1^H NMR (200 MHz, DMSO-d_6_): δ = 0.84 (s, 6H, C_Ad_H_3_), 1.02–1.18 (m, 2H, C_Ad_H_2_), 1.18–1.42 (m, 4H, C_Ad_H_2_), 1.46–1.71 (m, 4H, C_Ad_H_2_), 1.76–1.91 (m, 4H, C_Ad_H_2_), 2.15 (m, 1H, C_Ad_H), 2.38 (s, 3H, CH_3_), 2.95 (s, 3H, CH_3_N), 3.30–3.71 (m, 2H, CH_2_), 3.81–4.12 (m, 2H, CH_2_), 4.13–4.35 (m, 1H, CH_2_), 4.41–4.79 (m, 3H, CH_2_), 7.03 (d, 1H, ^3^*J*_HH_ = 8.3 Hz, C_Ar_H), 7.23 (s, 1H, C_Ar_H), 7.58 (d, 1H, ^3^*J*_HH_ = 8.3 Hz, C_Ar_H), 9.87 (br. S, 2H, NH), 11.26 (br.s, 1H, NH^+^CH_3_). ^13^C NMR (50 MHz, DMSO-d_6_): δ = 19.05 (CH_2_), 21.12 (CH_3_), 29.13 (CH), 29.66 (CH_3_), 32.02 (C), 36.05 (CH_2_), 37.70 (CH_2_), 39.04 (CH_2_), 41.37 (CH_3_), 41.52 (CH_2_), 43.26 (CH_2_), 49.49 (CH_2_), 50.05 (CH_2_), 57.90 (C), 101.59 (C), 109.59 (CH), 117.43 (CH), 123.35 (CH), 124.87 (C), 128.42 (C), 130.99 (C), 134.52 (C). Anal. C 67.47, H 8.79, N 8.58%. Calcd. For C_27_H_41_Cl_2_N_3_, C 67.77, H 8.64, N 8.78%.

*N*-(2-(8-Fluoro-2-methyl-3,4-dihydro-1*H*-pyrido[4,3-*b*]indol-5(2*H*)-yl)ethyl)-3,5-dimethyladamantane-1-amine dihydrochloride (**4c**). Brown solid. Yield 31%. Mp 215–216 °C. FT IR (KBr ν_max_ in cm^−1^): 3425 (NH), 2904 (CH_2_), 2848 (NCH_3_), 1611 (C=C), 1455 (CH of CH_2_). ^1^H NMR (200 MHz, DMSO-d_6_): δ = 0.85 (s, 6H, C_Ad_H_3_), 1.00–1.21 (m, 2H, C_Ad_H_2_), 1.21–1.43 (m, 4H, C_Ad_H_2_), 1.43–1.70 (m, 4H, C_Ad_H_2_), 1.70–1.90 (m, 2H, C_Ad_H_2_), 2.15 (m, 1H, C_Ad_H), 2.96 (s, 3H, CH_3_N), 3.15–3.37 (m, 2H, CH_2_), 3.38–3.70 (m, 4H, CH_2_), 3.98–4.34 (m, 1H, CH_2_), 4.38–4.75 (m, 3H, CH_2_), 7.06 (t, 1H, ^3^*J*_HF_ = ^3^*J*_HH_ = 9.3 Hz, C_Ar_H,), 7.31 (d, 1H, ^3^*J*_HH_ = 9.9 Hz, C_Ar_H,), 7.75 (dd, 1H, ^3^*J*_HH_ = 9.0 Hz, ^4^*J*_HF_ = 4.5 Hz, C_Ar_H,), 9.80 (s, 2H, NH), 11.26 (br.s, 1H, NH^+^CH_3_). ^13^C NMR (50 MHz, DMSO-d_6_): δ = 19.16 (CH_2_), 29.12 (CH), 29.65 (CH_3_), 32.07 (C), 36.04 (CH_2_), 37.70 (CH_2_), 41.39 (CH_3_), 41.51 (CH_2_), 43.26 (CH_2_), 49.45 (CH_2_), 49.90 (CH_2_), 57.95 (C), 102.30 (d, ^4^*J*_CF_ = 4.4 Hz, C), 103.08 (d, ^2^*J*_CF_ = 24.2 Hz, CH), 109.66 (d, ^2^*J*_CF_ = 25.6 Hz, CH), 110.95 (d, ^3^*J*_CF_ = 9.5 Hz, CH), 124.88 (d, ^3^*J*_CF_ = 10.6 Hz, C), 132.75 (C), 133.07 (C), 157.25 (d, ^1^*J*_CF_ = 233.1 Hz, CF). ^19^F NMR (188 MHz, DMSO-d_6_): δ = −124.4 (td, 1F, ^3^*J*_FH_ = 9.2 Hz, ^4^*J*_FH_ = 4.6 Hz, C_Ar_F). Anal. C 64.61, H 7.88, N 8.79%. Calcd. For C_26_H_38_Cl_2_FN_3_, C 64.72, H 7.94, N 8.71%.

*N*-(2-(2-Methyl-3,4-dihydro-1*H*-pyrido[4,3-*b*]indol-5(2*H*)-yl)ethyl)adamantane-1-amine dihydrochloride (**4d**). Pale brown solid. Yield 52%. Mp 160–162 °C. FT IR (KBr ν_max_ in cm^−1^): 3421 (NH), 2906 (CH_2_), 2852 (NCH_3_), 1614 (C=C), 1454 (CH of CH_2_). H NMR (200 MHz, DMSO-d_6_): δ = 1.37–1.68 (m, 6H, C_Ad_H_2_), 1.68–1.82 (m, 3H, C_Ad_H_2_), 1.82–1.96 (m, 3H, C_Ad_H_2_), 1.97–2.14 (m, 3H, C_Ad_H), 2.98 (d, 3H, ^3^*J*_HH_ = 4.4 Hz, CH_3_N), 3.15–3.37 (m, 3H, CH_2_), 3.38–3.58 (m, 1H, CH_2_), 3.74–3.96 (m, 1H, CH_2_), 3.82–4.14 (m, 2H, CH_2_), 4.27–4.48 (m, 1H, CH_2_), 4.54–4.84 (m, 2H, CH_2_), 7.39 (dd, 1H, ^3^*J*_HH_ = 8.4 Hz, ^3^*J*_HH_ = 7.9 Hz, C_Ar_H), 7.52 (dd, 1H, ^3^*J*_HH_ = 8.4 Hz, ^3^*J*_HH_ = 7.9 Hz, C_Ar_H), 7.78 (d, 1H, ^3^*J*_HH_ = 7.9 Hz, C_Ar_H), 7.97 (d, 1H, ^3^*J*_HH_ = 8.4 Hz, C_Ar_H), 8.29 (br. S, 2H, NH), 11.44 (br.s, 1H, NH^+^CH_3_). ^13^C NMR (50 MHz, DMSO): δ = 18.93 (CH_2_), 28.21 (CH), 28.34 (CH_2_), 35.04 (CH_2_), 37.44 (CH_2_), 41.24 (CH_3_), 49.32 (CH_2_), 49.88 (CH_2_), 50.84 (C), 102.04 (C), 109.74 (CH), 117.68 (CH), 119.63 (CH), 121.87 (CH), 124.55 (C), 130.96 (C), 136.01 (C). Anal. C 66.22, H 8.19, N 9.45%. Calcd. For C_24_H_35_Cl_2_N_3_, C 66.04, H 8.08, N 9.63%.

*N*-(2-(2,8-Dimethyl-3,4-dihydro-1*H*-pyrido[4,3-*b*]indol-5(2*H*)-yl)ethyl)adamantane-1-amine dihydrochloride (**4e**). Brown solid. Yield 43%. Mp 148–150 °C. FT IR (KBr ν_max_ in cm^−1^): 3412 (NH), 2914 (CH_2_), 2854 (NCH_3_), 1618 (C=C), 1464 (CH of CH_2_). ^1^H NMR (200 MHz, DMSO-d_6_): δ = 1.39–1.71 (m, 6H, C_Ad_H_2_), 1.71–1.85 (m, 3H, C_Ad_H_2_), 1.85–1.98 (m, 3H, C_Ad_H_2_), 1.98–2.16 (m, 3H, C_Ad_H), 2.34 (s, 3H, CH_3_), 2.93 (s, 3H, CH_3_N), 3.10–3.30 (m, 3H, CH_2_), 3.37–3.58 (m, 1H, CH_2_), 3.61–3.79 (m, 1H, CH_2_), 3.82–4.14 (m, 2H, CH_2_), 4.14–4.34 (m, 1H, CH_2_), 4.41–4.70 (m, 2H, CH_2_), 6.99 (d, 1H, ^3^*J*_HH_ = 8.6 Hz, C_Ar_H), 7.20 (s, 1H, C_Ar_H), 7.56 (d, 1H, ^3^*J*_HH_ = 8.6 Hz, C_Ar_H), 8.22 (br. S, 2H, NH), 11.34 (br.s, 1H, NH^+^CH_3_). ^13^C NMR (50 MHz, DMSO-d_6_): δ = 19.56 (CH_2_), 21.60 (CH_3_), 28.82 (CH), 28.94 (CH_2_), 35.65 (CH_2_), 37.93 (CH_2_), 41.35 (CH_3_), 49.95 (CH_2_), 50.50 (CH_2_), 51.45 (C), 102.13 (C), 110.04 (CH), 117.92 (CH), 123.78 (CH), 125.38 (C), 128.90 (C), 131.49 (C), 135.02 (C). Anal. C 66.51, H 8.47, N 9.46%. Calcd. For C_25_H_37_Cl_2_N_3_, C 66.65, H 8.28, N 9.33.

*N*-(2-(8-Fluoro-2-methyl-3,4-dihydro-1*H*-pyrido[4,3-*b*]indol-5(2*H*)-yl)ethyl)adamantane-1-amine dihydrochloride (**4f**). Brown solid. Yield 32%. Mp 214–215 °C. FT IR (KBr ν_max_ in cm^−1^): 3434 (NH), 2904 (CH_2_), 2846 (NCH_3_), 1610 (C=C), 1452 (CH of CH_2_). ^1^H NMR (200 MHz, DMSO-d_6_): δ = 1.42–1.72 (m, 6H, C_Ad_H_2_), 1.79 (br.s, 2H, C_Ad_H_2_), 1.79 (br.s, 4H, C_Ad_H_2_), 2.05 (s, 3H, C_Ad_H), 2.94 (s, 3H, NCH_3_), 2.96–3.29 (m, 4H, CH_2_), 3.26–3.78 (m, 3H, CH_2_), 4.14–4.34 (m, 1H, CH_2_), 4.45–4.69 (m, 2H, CH_2_), 7.05 (t, 1H, ^3^*J*_HF_ = ^3^*J*_HH_ = 9.0 Hz, C_Ar_H), 7.29 (d, 1H, ^3^*J*_HH_ = 9.0 Hz, ^4^*J*_HH_ = 2.3 Hz, C_Ar_H), 7.75 (dd, 1H, ^3^*J*_HH_ = 9.0 Hz, ^4^*J*_HF_ = 4.5 Hz, C_Ar_H), 8.17 (s, 2H, NH), 11.23 (br.s, 1H, NH^+^CH_3_). ^13^C NMR (50 MHz, DMSO-d_6_): δ = 19.10 (CH_2_), 28.30 (CH), 28.43 (CH), 35.13 (CH_2_), 35.21 (CH_2_), 37.47 (CH_2_), 39.65 (CH_2_), 41.30 (CH_3_), 49.29 (CH_2_), 49.43 (CH_2_), 50.92 (C), 102.33 (d, ^4^*J*_CF_ = 4.4 Hz, C), 102.13 (d, ^2^*J*_CF_ = 24.2 Hz, CH), 109.71 (d, ^2^*J*_CF_ = 26.0 Hz, CH), 110.98 (d, ^3^*J*_CF_ = 9.5 Hz, CH), 124.94 (d, ^3^*J*_CF_ = 10.6 Hz, C), 132.79 (C), 133.13 (C), 157.29 (d, ^1^*J*_CF_ = 233.1 Hz, CF). ^19^F NMR (188 MHz, DMSO-d_6_): δ = −124.8 (td, 1F, ^3^*J*_FH_ = 9.2 Hz, ^4^*J*_FH_ = 4.6 Hz, CF). Anal. C 63.57, H 7.43, N 9.14%. Calcd. For C_24_H_34_Cl_2_FN_3_, C 63.43, H 7.54, N 9.25%.

#### 3.1.2. General Procedure for the Preparation of Compounds **8a**–**f**

A mixture of carboline **5a**–**c** (1 mmol), chloroacetamide **6** (1 mmol) and KOH (1.1 mmol) in DMF (10 mL) was stirred at 20 °C for 3 h. The reaction mixture was poured into water (50 mL). The resulting solid was filtered off, dried and dissolved in acetone (10 mL). A 37% (*v*/*v*) aqueous HCl (0.2 mL) was added to this solution and the precipitate that formed was filtered off and dried.

3,5-Dimethyladamantan-1-yl)-2-(2-methyl-3,4-dihydro-1*H*-pyrido[4,3-*b*]indol-5(2*H*)-yl)acetamide hydrochloride (**8a**). White solid. Yield 63%, mp 257–259 °C. FT IR (KBr ν_max_ in cm^−1^): 3410 (NH), 3260 (CONH), 2904 (CH_2_), 2852 (NCH_3_), 1676 (C=O), 1542 (NH of amide), 1455 (CH of CH_2_). ^1^H NMR (200 MHz, DMSO-d_6_): δ = 0.85 (s, 6H, C_Ad_H_3_), 1.01–1.22 (m, 2H, C_Ad_H_2_), 1.22–1.43 (m, 4H, C_Ad_H_2_), 1.51–1.75 (m, 4H, C_Ad_H_2_), 1.76–1.91 (m, 2H, C_Ad_H_2_), 2.16 (br.s, 1H, C_Ad_H), 2.96 (d, 3H, ^3^*J*_HH_ = 4.0 Hz, CH_3_N), 3.05–3.30 (m, 2H, CH_2_), 3.30–3.80 (m, 2H, CH_2_), 4.06–4.60 (m, 2H, CH_2_), 4.75 (s, 2H, CH_2_), 6.90–7.26 (m, 2H, CH_Ar_), 7.28–7.62 (m, 2H, CH_Ar_), 8.21 (s, 1H, NH), 11.38 (br.s, 1H, NH^+^CH_3_). ^13^C NMR (50 MHz, DMSO-d_6_): δ = 19.26 (CH_2_), 29.45 (CH), 30.03 (CH_3_), 31.80 (C), 39.41 (CH_2_), 41.44 (CH_2_), 42.22 (CH_3_), 45.99 (CH_2_), 46.85 (CH_2_), 49.44 (CH_2_), 49.95 (CH_2_), 50.16 (CH_2_), 52.70 (C), 103.33 (C), 109.68 (CH), 117.43 (CH), 119.29 (CH), 121.37 (CH), 124.33 (CH), 131.95 (C), 136.86 (C), 166.31 (C=O). Anal. C 70.45, H 8.12, N 9.27%. Calcd. For C_26_H_36_ClN_3_O, C 70.65, H 8.21, N 9.51%.

2-(2,8-Dimethyl-3,4-dihydro-1*H*-pyrido[4,3-*b*]indol-5(2*H*)-yl)-*N*-(3,5-dimethyladamantan-1-yl)acetamide hydrochloride (**8b**). White solid. Yield 54%. Mp 205–208 °C. FT IR (KBr ν_max_ in cm^−1^): 3421 (NH), 3254 (CONH), 2902 (CH_2_), 2861 (NCH_3_), 1678 (C=O), 1543 (NH of amide), 1466 (CH of CH_2_). ^1^H NMR (200 MHz, DMSO-d_6_): δ = 0.77 (s, 6H, C_Ad_H_3_), 1.00–1.12 (m, 2H, C_Ad_H_2_), 1.12–1.39 (m, 4H, C_Ad_H_2_), 1.44–1.66 (m, 4H, C_Ad_H_2_), 1.66–1.84 (m, 2H, C_Ad_H_2_), 2.04 (br.s, 1H, C_Ad_H), 2.35 (s, 3H, CH_3_), 2.89 (s, 3H, CH_3_N), 2.98–3.24 (m, 2H, CH_2_), 3.36–3.78 (m, 2H, CH_2_), 4.08–4.59 (m, 2H, CH_2_), 4.68 (s, 2H, CH_2_), 6.95 (d, 1H, ^3^*J*_HH_ = 8.1 Hz, C_Ar_H), 7.18 (s, 1H, C_Ar_H), 7.31 (d, 1H, ^3^*J*_HH_ = 8.1 Hz, C_Ar_H), 8.06 (s, 1H, NH), 11.18 (br.s, 1H, NH^+^CH_3_). ^13^C NMR (50 MHz, DMSO-d_6_): δ = 19.24 (CH_2_), 21.10 (CH_3_), 29.43 (CH), 30.01 (CH_3_), 31.80 (C), 39.40 (CH_2_), 41.46 (CH_3_), 42.20 (CH_2_), 46.04 (CH_2_), 46.86 (CH_2_), 49.54 (CH_2_), 50.06 (CH_2_), 50.14 (CH_2_), 52.67 (C), 100.82 (C), 109.34 (CH), 117.12 (CH), 122.85 (CH), 124.54 (C), 127.91 (C), 131.90 (C), 135.30 (C), 166.32 (C=O). Anal. C 71.23, H 8.24, N 9.29%. Calcd. For C_27_H_38_ClN_3_O, C 71.11, H 8.40, N 9.21%.

*N*-(3,5-Dimethyladamantan-1-yl)-2-(8-fluoro-2-methyl-3,4-dihydro-1*H*-pyrido[4,3-*b*]indol-5(2*H*)-yl)acetamide hydrochloride (**8c**). White solid. Yield 68%. Mp 256–258 °C. FT IR (KBr ν_max_ in cm^−1^): 3447 (NH), 3274 (CONH), 2898 (CH_2_), 2841 (NCH_3_), 1677 (C=O), 1545 (NH of amide), 1453 (CH of CH_2_). ^1^H NMR (200 MHz, DMSO-d_6_): δ = 0.80 (s, 6H, C_Ad_H_3_), 1.00–1.15 (m, 2H, C_Ad_H_2_), 1.15–1.40 (m, 4H, C_Ad_H_2_), 1.46–1.69 (m, 4H, C_Ad_H_2_), 1.69–1.87 (m, 2H, C_Ad_H_2_), 1.97–2.20 (m, 1H, C_Ad_H), 2.96 (d, 3H, ^3^*J*_HH_ = 4.0 Hz, CH_3_N), 3.05–3.30 (m, 2H, CH_2_), 3.36–3.60 (m, 2H, CH_2_), 4.25 (dd, 1H, ^2^*J*_HH_ = 13.4 Hz, ^3^*J*_HH_ = 6.3 Hz, CH_2_), 4.58 (d, 1H, ^2^*J*_HH_ = 13.4 Hz, CH_2_), 4.75 (s, 2H, CH_2_), 7.01 (td, 1H, ^3^*J*_HF_ = ^3^*J*_HH_ = 9.3 Hz, ^4^*J*_HH_ = 2.3 C_Ar_H,), 7.26 (dd, 1H, ^3^*J*_HH_ = 9.9 Hz, ^4^*J*_HH_ = 2.3 Hz, C_Ar_H,), 7.45 (dd, 1H, ^3^*J*_HH_ = 9.0 Hz, ^4^*J*_HF_ = 4.5 Hz, C_Ar_H,), 8.07 (s, 1H, NH), 10.82 (br.s, 1H, NH^+^CH_3_). ^13^C NMR (50 MHz, DMSO-d_6_): δ = 19.28 (CH_2_), 29.33 (CH), 29.91 (CH_3_), 31.70 (C), 39.29 (CH_2_), 41.37 (CH_3_), 42.10 (CH_2_), 46.10 (CH_2_), 46.74 (CH_2_), 49.25 (CH_2_), 49.78 (CH_2_), 50.04 (CH_2_), 52.63 (C), 101.54 (d, ^4^*J*_CF_ = 4.4 Hz, C), 102.60 (d, ^2^*J*_CF_ = 23.8 Hz, CH), 109.09 (d, ^2^*J*_CF_ = 26.0 Hz, CH), 110.63 (d, ^3^*J*_CF_ = 9.1 Hz, CH), 124.44 (d, ^3^*J*_CF_ = 10.2 Hz, C), 133.48 (C), 133.91 (C), 156.97 (d, ^1^*J*_CF_ = 232.4 Hz, CF), 166.03 (C=O). ^19^F NMR (188 MHz, DMSO-d_6_): δ = −126.2 (td, 1F, ^3^*J*_FH_ = 9.2 Hz, ^4^*J*_FH_ = 4.6 Hz, CF). Anal. C 67.73, H 7.44, N 9.24%. Calcd. For C_26_H_35_ClFN_3_O, C 67.88, H 7.67, N 9.13%.

*N*-(Adamantan-1-yl)-2-(2-methyl-3,4-dihydro-1*H*-pyrido[4,3-*b*]indol-5(2*H*)-yl)acetamide hydrochloride (**8d**). Grey solid. Yield 64%. Mp 246–248 °C. FT IR (KBr ν_max_ in cm^−1^): 3376 (NH), 3256 (CONH), 2908 (CH_2_), 2849 (NCH_3_), 1679 (C=O), 1542 (NH of amide), 1466 (CH of CH_2_). ^1^H NMR (200 MHz, DMSO-d_6_): δ = 1.58 (br.s, 6H, C_Ad_H_2_), 1.92 (br.s, 6H, C_Ad_H_2_), 1.96 (br.s, 3H, C_Ad_H), 2.89 (d, 3H, ^3^*J*_HH_ = 3.5 Hz, NCH_3_), 2.96–3.28 (m, 2H, CH_2_), 3.28–3.78 (m, 2H, CH_2_), 4.23 (dd, 1H, ^2^*J*_HH_ = 14.0 Hz, ^3^*J*_HH_ = 6.3 Hz, CH_2_), 4.05 (d, 1H, ^2^*J*_HH_ = 14.0 Hz, CH_2_), 4.74 (s, 2H, CH_2_C(O)), 6.95–7.21 (m, 2H, CH_Ar_), 7.33–7.55 (m, 2H, CH_Ar_), 8.11 (s, 1H, NH), 11.28 (br.s, 1H, NH^+^CH_3_). ^13^C NMR (50 MHz, DMSO-d_6_): δ = 19.25 (CH_2_), 28.75 (CH), 35.94 (CH_2_), 40.90 (CH_2_), 41.47 (CH_3_), 46.02 (CH_2_), 49.49 (CH_2_), 49.99 (CH_2_), 51.15 (C), 101.38 (C), 109.68 (CH), 117.44 (CH), 119.30 (CH), 121.38 (CH), 124.33 (C), 131.95 (C), 136.90 (C), 166.23 (C=O). Anal. C 69.52, H 7.63, N 10.23%. Calcd. For C_24_H_32_ClN_3_O, C 69.63, H 7.79, N 10.15%.

*N*-(Adamantan-1-yl)-2-(2,8-dimethyl-3,4-dihydro-1*H*-pyrido[4,3-*b*]indol-5(2*H*)-yl)acetamide hydrochloride (**8e**). Pale brown solid. Yield 72%. Mp 260–262 °C. FT IR (KBr ν_max_ in cm^−1^): 3425 (NH), 3271 (CONH), 2907 (CH_2_), 2850 (NCH_3_), 1675 (C=O), 1542 (NH of amide), 1455 (CH of CH_2_). ^1^H NMR (200 MHz, DMSO-d_6_): δ = 1.60 (br.s, 6H, C_Ad_H_2_), 1.93 (br.s, 6H, C_Ad_H_2_), 1.98 (br.s, 3H, C_Ad_H), 2.37 (s, 3H, CH_3_), 2.92 (d, 3H, ^3^*J*_HH_ = 3.5 Hz, NCH_3_), 2.96–3.25 (m, 2H, CH_2_), 3.26–3.78 (m, 2H, CH_2_), 4.07–4.35 (m, 1H, CH_2_), 4.05 (d, 1H, ^2^*J*_HH_ = 12.6 Hz, CH_2_), 4.76 (s, 2H, CH_2_C(O)), 6.94 (d, 1H, ^3^*J*_HH_ = 8.1 Hz, C_Ar_H), 7.18 (s, 1H, C_Ar_H), 7.32 (d, 1H, ^3^*J*_HH_ = 8.6 Hz, C_Ar_H), 8.04 (br. S, 1H, NH), 11.23 (br.s, 1H, NH^+^CH_3_). ^13^C NMR (50 MHz, DMSO-d_6_): δ = 19.19 (CH_2_), 21.03 (CH_3_), 28.68 (CH), 35.88 (CH_2_), 40.33 (CH_2_), 41.40 (CH_3_), 46.03 (CH_2_), 49.47 (CH_2_), 49.97 (CH_2_), 51.06 (C), 100.77 (C), 109.33 (CH), 117.06 (CH), 122.76 (CH), 124.48 (C), 127.83 (C), 131.87 (C), 135.27 (C), 166.21 (C=O). Anal. C 70.24, H 8.21, N 9.74%. Calcd. For C_25_H_34_ClN_3_O, C 70.15, H 8.01, N 9.82%.

*N*-(Adamantan-1-yl)-2-(8-fluoro-2-methyl-3,4-dihydro-1*H*-pyrido[4,3-*b*]indol-5(2*H*)-yl)acetamide hydrochloride (**8f**). Pale brown solid. Yield 74%. Mp 230–232 °C. FT IR (KBr ν_max_ in cm^−1^): 3406 (NH), 3263 (CONH), 2910 (CH_2_), 2849 (NCH_3_), 1679 (C=O), 1542 (NH of amide), 1460 (CH of CH_2_). ^1^H NMR (200 MHz, DMSO-d_6_): δ = 1.68 (br.s, 6H, C_Ad_H_2_), 1.91 (br.s, 9H, C_Ad_H + C_Ad_H_2_), 2.88 (s, 3H, NCH_3_), 2.91–3.30 (m, 2H, CH_2_), 3.36–3.82 (m, 2H, CH_2_), 4.35 (s, 2H, CH_2_), 4.76 (s, 2H, CH_2_C(O)), 6.96 (t, 1H, ^3^*J*_HF_ = ^3^*J*_HH_ = 9.0 Hz, C_Ar_H,), 7.22 (d, 1H, ^3^*J*_HH_ = 8.5 Hz, C_Ar_H,), 7.47 (dd, 1H, ^3^*J*_HH_ = 9.0 Hz, ^4^*J*_HF_ = 4.5 Hz, C_Ar_H,), 8.15 (s, 1H, NH), 11.32 (br.s, 1H, NH^+^CH_3_). ^13^C NMR (50 MHz, DMSO-d_6_): δ = 19.33 (CH_2_), 28.69 (CH), 35.87 (CH_2_), 40.82 (CH_2_), 41.41 (CH_3_), 46.16 (CH_2_), 49.29 (CH_2_), 49.87 (CH_2_), 51.12 (C), 101.60 (d, ^4^*J*_CF_ = 4.4 Hz, C), 102.67 (d, ^2^*J*_CF_ = 24.5 Hz, CH), 109.12 (d, ^2^*J*_CF_ = 26.0 Hz, CH), 110.71 (d, ^3^*J*_CF_ = 9.5 Hz, CH), 124.48 (d, ^3^*J*_CF_ = 10.2 Hz, C), 133.56 (C), 133.93 (C), 157.03 (d, ^1^*J*_CF_ = 232.0 Hz, CF), 166.01 (C=O). ^19^F NMR (188 MHz, DMSO-d_6_): δ = −116.9 (td, 1F, ^3^*J*_FH_ = 8.5 Hz, ^4^*J*_FH_ = 4.5 Hz, CF). Anal. C 66.70, H 7.41, N 9.92%. Calcd. For C_24_H_31_ClFN_3_O, C 66.73, H 7.23, N 9.73%.

### 3.2. Evaluation of the Effect of New Compounds on NMDA-Receptors by a Radioligand Binding Assay

The effect of the test compounds on radioligand binding to NMDA receptors was evaluated by a modified method, as previously reported [83]. The following two radioligands were used: [^3^H] MK-801 (dizocilpine) with a specific activity of 210 Ci/mmol, binding to all isolated NMDA receptors; and [^3^H] ifenprodil with a specific activity of 79 Ci/mmol, binding only to NMDA receptors containing the NR2B subunit [84,85]. A hippocampal membrane preparation for the radioligand binding assay was made as described previously [86,87]. The obtained membrane pellet was resuspended in a working buffer (5 mM HEPES/4.5 mM Tris, pH 7.6 at 4 °C) in a ratio of 1:5 and stored in liquid nitrogen. The reaction mixture (the final volume was 0.5 mL) contained the working buffer (200 μL), a 50 nM radioligand solution (50 μL) and the membrane suspension (250 μL). The nonspecific binding was evaluated in the presence of 1 mM of the unlabeled ligand (50 μL).

For the binding assay, the reaction mixture was incubated at room temperature for 2 h. After the incubation, the samples were filtered through GF/B glass fiber filters (Whatman), washed with the working buffer, dried and transferred to scintillation vials. Then the scintillation fluid (5 mL), which is composed of diphenyloxazole (PPO) (4 g) and bis(5-phenyl-2-oxazolyl)benzene (POPOP) (0.2 g) in toluene (1 L) were added to the vials. The radioactivity was measured with a TriCarb2800 TR scintillation counter (Perkin Elmer, Waltham, MA, USA) with a counting efficiency of about 65%.

The effect of the test compounds on the binding of [^3^H] MK-801 and [^3^H] ifenprodil to rat hippocampal membranes was evaluated by the addition of the test compounds (50 μL) to the incubation medium in the concentration range of 10^−8^–10^−3^ M. The IC_50_ values for the test compounds were calculated from the inhibition data with GraphPad Prism 4 (Supplement Figure S5). The values of *K*_i_ were calculated according to a previously described method [88].

### 3.3. Analysis of the Esterase Profile of Compounds and Inhibition Kinetics of AChE and BChE

Kinetic studies were performed using commercial human erythrocyte AChE, equine serum BChE and porcine liver CES (Sigma, USA). The AChE and BChE activity was evaluated by Ellman’s method (λ = 412 nm) [89] using 1 mM acetylthiocholine and 1 mM butyrylthiocholine (Sigma, St. Louis, MO, USA), respectively, as the substrate in 100 mM phosphate buffer, pH 7.5, 0.33 mM DTNB, 0.02 unit/mL of AChE or BChE at 25 °C as described in detail in [71]. Reagents blanks consisted of reaction mixtures without substrates. The CES activity was measured spectrophotometrically based on the release of 4-nitrophenol (λ = 405 nm) in the presence of 1 mM 4-nitrophenyl acetate as the substrate [90] in 100 mM phosphate buffer, pH 8.0, at 25 °C as described in [71]. Final enzyme and substrate concentrations were 0.02 unit/mL and 1 mM, respectively. Assays were carried out with a blank containing all constituents except porcine CES to assess non-enzymatic hydrolysis. The measurements were performed on a FLUOstar OPTIMA multifunctional microplate reader (BMG LABTECH, Ortenberg, Germany). The compounds were dissolved in DMSO. The incubation mixture contained 2% (*v*/*v*) of the solvent. The primary inhibitory activity of the test compounds was determined by the measurement of the degree of inhibition of the enzymes with a 20 µM concentration of the compound. The sample of the appropriate enzyme was incubated with the test compound for 5 min, and then the residual enzymatic activity was determined. Each experiment was performed in triplicate. The IC_50_ values, i.e., the concentration of the compounds required for 50% inhibition of the enzyme, were determined for the most active compounds. To measure the IC_50_ values of AChE, BChE and CES inhibition, the samples of the enzymes were incubated with the test compound in the concentration range of 1 × 10^–12^–1 × 10^–4^ M for 5 min, and then the residual enzymatic activity was evaluated. Each experiment was performed in triplicate. The IC_50_ values were calculated with Origin 6.1 software. Tacrine, an effective AChE and BChE inhibitor, and bis-4-nitrophenyl phosphate (BNPP), a selective CES inhibitor, were employed as the positive controls.

The mechanisms of AChE and BChE inhibition were assessed by a thorough analysis of enzyme kinetics. The residual activity was determined after a 5-min incubation at 25 °C (for temperature equilibration) with three increasing concentrations of the inhibitor and six decreasing substrate concentrations. The inhibition constants *K*_i_ (a competitive component) and α*K*_i_ (a noncompetitive component) were determined by the linear regression of 1/V versus 1/[S] double-reciprocal (Lineweaver-Burk) plots.

### 3.4. Displacement of Propidium Iodide from AChE PAS

The compounds were evaluated as potential inhibitors of proaggregation activity of AChE by measuring the displacement of propidium iodide from the PAS of the enzyme [53] using a fluorescence method [28]. AChE from *Electrophorus electricus* (*Ee*AChE, type VI-S, lyophilized powder, Sigma-Aldrich, St. Louis, MO, USA) was used for consistency with our previous studies [8,13,63,74]. The fluorescence intensity of propidium iodide bound to AChE increases several-fold. A decrease in the fluorescence intensity in the presence of the test compounds is indicative of their ability to bind to the AChE PAS and, correspondingly, to inhibit β-amyloid aggregation.

*Ee*AChE (7 µM) was incubated with 20 µM of the test compound in 1 mM Tris-HCl buffer, pH 8.0, at 25 °C for 15 min. Then a solution of propidium iodide was added (final concentration 8 µM), the mixture was incubated for 15 min, and the fluorescence spectrum was recorded (excitation at 530 nm and emission at 600 nm). Donepezil and tacrine were used as the reference compounds. The blank contained propidium iodide at the same concentration in 1 mM Tris-HCl buffer, pH 8.0 at 25 °C. The measurements were performed in triplicate on a FLUOstar OPTIMA multifunctional microplate reader.

The degree of displacement of propidium iodide from the PAS of AChE (% displacement) was calculated by the equation:

% displacement = 100 − (IF_AChE + Propidium + inhibitor_/IF_AChE + Propidium_) × 100,

where IF_AChE + Propidium_ is the fluorescence intensity of propidium iodide bound to *Ee*AChE in the absence of the test compound (taken as 100%), and IF_AChE + Propidium + inhibitor_ is the fluorescence intensity of propidium iodide bound to *Ee*AChE in the presence of the test compound.

### 3.5. Investigation of the Effect of the Compounds on Mitochondrial Characteristics

Experiments were performed using outbred male rats weighing 200–220 g. The animals were housed under standard vivarium conditions in a normal day/night cycle and had *ad libitum* access to water and food. The procedure of rat euthanasia using carbon dioxide (CO_2_) inhalation for mitochondria preparation is in compliance with the Guidelines for Animal Experiments, which were approved by the local bioethics committee of the IPAC RAS.

Rat liver mitochondria were isolated by conventional differential centrifugation [91]. Membrane potential of rat liver mitochondria was measured using the potential-sensitive fluorescent probe Safranine A on a Victor 3 multilabel plate reader (Perkin Elmer, Waltham, MA, USA). The fluorescence was recorded at λ_ex_ = 485 nm and λ_em_ = 590 nm [92]. Mitochondrial swelling was measured by spectrophotometry in 96-well clear bottom plates on a Victor 3 microplate reader (Perkin Elmer, Waltham, MA, USA) at 620 nm; CaCl_2_ was added to induce mitochondrial swelling (MPT opening) [20].

### 3.6. MTT Assay

The MTT assay is commonly used to evaluate cell viability after exposure to toxic substances and is based on the ability of dehydrogenases of living cells, in particular, succinate dehydrogenase, to reduce colorless 3-(4,5-dimethylthiazol-2-yl)-2–5-diphenyltetrazolium salts (MTT reagent) to blue crystalline formazan, which is soluble in dimethyl sulfoxide. The MTT assay was performed by a slightly modified procedure [93] after a 24-h incubation of SH-SY5Y cells with the test compound at a concentration of 30 µM. The concentration of reduced product was measured colorimetrically on a Victor 3 microplate reader (Perkin Elmer, USA) based on the difference in absorbance of the sample at wavelengths of 540–620 nm.

### 3.7. Evaluation of the Effect of Compounds on Tubulin Polymerization

A crude fraction of tubulin and microtubule-associated proteins (Tb+MAP) was isolated from mouse brain tissue by a polymerization–depolymerization method [94]. The procedure of mouse euthanasia using cervical dislocation for Tb+MAP preparation is in compliance with the Guidelines for Animal Experiments, which were approved by the local bioethics committee of the IPAC RAS. The freshly dissected brain was immediately placed on ice, cleared of meninges and surface blood vessels, and washed with cold buffer A (50 mM Tris-HCl, pH 6.9 at 4 °C, 2 mM EGTA). The extracted brain tissue was homogenized under ice cooling in the same buffer using a Potter S homogenizer (Sartorius). The resulting homogenate was centrifuged at 10,000× *g* for 30 min on an Avanti 25 centrifuge (Beckman) using a JA-14 rotor for the precipitation of non-ruptured cells. The precipitate was discarded, and the supernatant was again centrifuged at 100,000× *g* for 60 min at 4 °C on an Optima MAX XP centrifuge (Beckman) using a MLA-50 rotor. The precipitate was again discarded. The protein concentration in the supernatant was determined by the Bradford assay using the Bio-Rad Protein Assay kit (Bio-Rad, Hercules, CA, USA) according to manufacturer’s instructions. The resulting supernatant was a cytosolic fraction enriched in microtubular proteins (Tb and MAP).

The Tb+MAP preparation was centrifuged at 5000× *g* for 10 min at 4 °C immediately before polymerization in order to remove denatured and aggregated protein molecules by precipitation. The Tb+MAP polymerization was performed at 37 °C in buffer A in the presence of 0.1 mM GTP after addition of 100 µM of test compound or the same volume of vehicle (DMSO). The protein concentration in the sample was 0.2 mg/mL. The polymerization kinetics was monitored on a Victor 3 or EnVision microplate reader (Perkin Elmer, USA) at 355 nm and the stabilized rate of polymerization after the first wave was measured (dA_355_/dt).

### 3.8. Molecular Modeling

To prepare the structures for molecular modeling, the protonation state of compounds was selected based on the estimated p*K*_a_ of ionogenic groups with Marvin 16.6.13.0 software (ChemAxon, http://www.chemaxon.com (accessed on 23 August 2021)). Geometry optimization of the ligands was carried out using GAMESS-US software [95] using DFT functional B3LYP with the 6–31G* basis set. Molecular docking of ligands into the active sites of AChE and BChE was performed using crystal structures PDB ID 4EY4 [66] and PDB ID 1P0I [96], respectively. The protein structure was prepared, saturated with water molecules, and optimized by the QM/MM method as reported previously [97]. Molecular docking was carried out with the Lamarckian genetic algorithm (LGA) [98] implemented in Autodock software 4.2.6 [99]. The grid box for docking included the entire active site gorge of AChE (22.5 Å × 22.5 Å × 22.5 Å grid box dimensions) and BChE (15 Å × 20.25 Å × 18 Å grid box dimensions) with a grid spacing of 0.375 Å. The following LGA parameters were used: 256 runs, 27 × 10^4^ generations, population size 3000 and 25 × 10^6^ evaluations.

## 4. Conclusions

All of the new compounds exhibited activity in the primary screening assays used in this study and possessed a multifunctional type of action. Thus, the compounds inhibited AChE and BChE, demonstrating stronger inhibitory activity against BChE, but had almost no effect on the activity of CES, which is an anti-target for anticholinesterase agents. The compounds were NMDA-subtype glutamate receptor ligands, exhibited mitoprotective properties, increased the resistance of mitochondria to the MPT pore opening and acted as microtubule stabilizers stimulating the polymerization of tubulin and microtubule-associated proteins. A new property of the conjugates, which is absent in the agents containing only one pharmacologically active scaffold (memantine and dimebon), was revealed: the ability to occupy the PAS of AChE, which could confer a disease-modifying effect of blocking AChE-induced β-amyloid aggregation.

Unexpected results were obtained in the study of the effect of the compounds on NMDA receptors. In particular, the conjugates containing memantine, the specific ligand of the intra-channel site of the NMDA receptor, exhibited activity against this site comparable with that of the conjugates with amantadine, which itself does not show any notable activity toward this target. In general, the modification of the polycyclic scaffolds in the tested conjugates did not have a significant effect on the activity of the compounds. It should be emphasized that the synthesized conjugates exhibited a new type of activity compared to the prototypes (dimebon and memantine)—the ability to block the ifenprodil-binding site of the NMDA receptor.

The spacer structure had a substantial effect on the activity of the compounds. In all cases, except for the stimulation of tubulin polymerization, the use of the ethylene spacer instead of the 1-oxoethylene one significantly enhanced interactions with the studied targets. Namely, this spacer configuration improved the binding of the conjugates to both sites of the NMDA receptor, increased the anti-BChE activity and enhanced the ability to inhibit MPT pore opening. In addition, the conjugates linked via the ethylene spacer could moderately inhibit AChE and block AChE-induced β-amyloid aggregation. In the series of new compounds, there were several hits, where conjugates linked via the ethylene spacer were revealed, and in this series, several potential lead compounds (**4a**, **4c**, **4d**, **4f**) were selected. Taking into account the efficacy of these compounds toward the selected targets, they would be expected to have cognitive-enhancing, neuroprotective, anti-amyloid and microtubule-stabilizing activity. Therefore, the investigation of the newly synthesized conjugated structures bearing scaffolds of the known neuroactive agents (memantine and dimebon) resulted in the synthesis of a novel group of multipharmacophore compounds and revealed unexpected properties that were not inherent to the background “monopharmacophore” agents. As well as previously described aminoadamantane-carbazole derivatives [9], the newly synthesized conjugates look highly promising as potential multifunctional agents that act simultaneously on a number of key biological targets involved in the pathogenesis of neurodegenerative processes. Preclinical trials of safety and tolerance of lead compounds could permit the selection of the most promising drug candidates for further development.

## Figures and Tables

**Table 1:** Binding of compounds to MK-801-specific and Ifenprodil-specific sites of NMDA receptors.

Compound	R	*K*_i_, µM, MK801-Binding Site	*K*_i_, µM, Ifenprodil-Binding Site
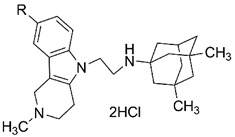			
**4a**	H	1.9 ± 0.2	1.7 ± 0.2
**4b**	Me	7.9 ± 0.8	8.8 ± 1.0
**4c**	F	4.7 ± 0.5	3.1 ± 0.3
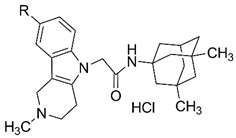			
**8a**	H	63.5 ± 5.8	112.7 ± 10.4
**8b**	Me	55.1 ± 5.1	89.7 ± 8.7
**8c**	F	80.6 ± 8.5	110.2 ± 10.8
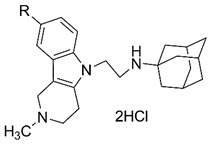			
**4d**	H	3.3 ± 0.3	4.4 ± 0.3
**4e**	Me	10.3 ± 1.6	8.2 ± 0.9
**4f**	F	5.4 ± 0.6	5.1 ± 0.5
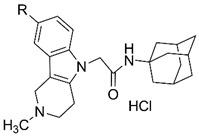			
**8d**	H	49.5 ± 4.5	79.1 ± 7.1
**8e**	Me	72.7 ± 6.6	104.5 ± 10.0
**8f**	F	47.3 ± 4.7	73.8 ± 7.0
**Reference Agents**			
**dimebon**		50.4 ± 4.3	77.2 ± 5.7
**memantine**		0.8 ± 0.1	115.1 ± 6.5

Data are expressed as mean ± SEM (*n* = 3).

**Table 2:** Esterase profiles of aminoadamantane–γ-carboline conjugates and their ability to displace propidium iodide from the AChE PAS.

No	R	IC_50_ (µM) or % of Inhibition of the Enzyme with 20 µM Concentration of the Compound			% of Displacement of Propidium Iodide from the PAS of *Ee*AChE with 20 µM Concentration of the Compound
		**AChE**	**BChE**	**CES**	
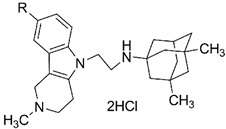					
**4a**	H	19.5 ± 0.3%	0.666 ± 0.037	15.6 ± 0.3%	12.5 ± 1.1%
**4b**	Me	50.3 ± 3.4	5.07 ± 0.09	10.4 ± 0.5%	14.7 ± 0.9%
**4c**	F	39.7 ± 3.2	0.729 ± 0.011	6.9 ± 0.4%	13.1 ± 1.0%
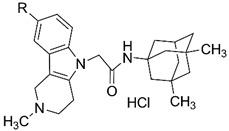					
**8a**	H	7.1 ± 0.5%	9.66 ± 0.52	5.2 ± 1.2%	n.a.
**8b**	Me	17.7 ± 1.4%	33.7 ± 3.5	6.4 ± 0.5%	2.8 ± 0.2%
**8c**	F	17.3 ± 0.8%	6.00 ± 0.06	8.4 ± 0.9%	n.a.
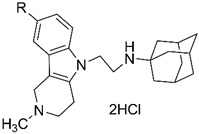					
**4d**	H	112 ± 5	2.48 ± 0.22	2.9 ± 0.8%	10.1 ± 0.9%
**4e**	Me	63.3 ± 3.3	9.05 ± 0.80	2.5 ± 0.5%	15.1 ± 1.2%
**4f**	F	13.6 ± 1.3	2.53 ± 0.10	8.6 ± 1.7%	17.8 ± 1.5%
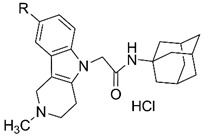					
**8d**	H	5.4 ± 1.0%	26.5 ± 0.4	8.8 ± 1.2%	1.4 ± 0.2%
**8e**	Me	13.5 ± 0.9%	43.2 ± 2.9	9.9 ± 1.5%	1.2 ± 0.1%
**8f**	F	22.1 ± 1.8%	17.1 ± 1.3	6.2 ± 0.6%	n.a.
**Reference Agents**					
**dimebon**		36.3 ± 3.6	5.76 ± 0.51	n.a.	4.1 ± 0.3%
**memantine**		1.3 ± 0.2%	13.7 ± 1.5%	3.0 ± 0.4%	n.a.
**donepezil**		0.040 ± 0.004	19.2 ± 2.0	>100	11.9 ± 0.9%
**tacrine**		0.60 ± 0.05	0.029 ± 0.002	n.a.	4.2 ± 0.3%
**BNPP**		n.a.	n.a.	1.80 ± 0.11	n.e.

n.a. = not active. n.e. = not evaluated. Data are expressed as mean ± SEM, *n* = 3.

**Table 3:** Effects of compounds on the calcium-induced MPT pore opening and the rate of GTP-dependent Tb+MAP polymerization.

Compound	R	Rate of Ca^2+^-Induced Mitochondrial Swelling (dA_620_/dt), % *	Rate of Tb+MAP Polymerization (dA_355_/dt), % **
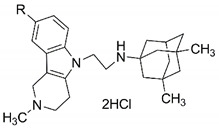			
**4a**	H	33 ± 4	126 ± 9
**4b**	Me	39 ± 7	153 ± 33
**4c**	F	31 ± 10	166 ± 11
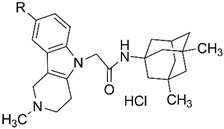			
**8a**	H	42 ± 21	237 ± 55
**8b**	Me	87 ± 33	314 ± 46
**8c**	F	63 ± 25	359 ± 25
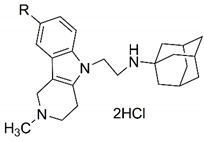			
**4d**	H	35 ± 13	162 ± 19
**4e**	Me	42 ± 9	239 ± 21
**4f**	F	42 ± 8	356 ± 14
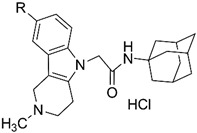			
**8d**	H	45 ± 17	128 ± 15
**8e**	Me	49 ± 12	133 ± 28
**8f**	F	61 ± 24	154 ± 14
**Reference Agents**			
**dimebon**		36 ± 11	162 ± 23
**memantine**		70 ± 9	89 ± 7

* The effect of 30 µM compounds on the maximum rate of Ca^2+^-induced swelling of isolated mitochondria (dA_620_/dt), %). Data are presented as the percentage relative to the reference compound; the Ca^2+^ concentration was 12.5 µM; the following mixture of substrates was used: 10 mM glutamate, 2 mM malate and 10 mM succinate. ** The effect of 100 µM compounds on the rate of Tb+MAP polymerization (dA_355_/dt), %).

## Supplementary Materials

The following are available online: Table S1, Docking scores of aminoadamantane–γ-carboline conjugates; Figure S1, IC_50_ values for hAChE inhibition by compounds **4** (MEAN ± SEM, *n* = 3); Figure S2, IC_50_ values for eqBChE inhibition by compounds **4**, **8** (MEAN ± SEM, *n* = 3); Figure S3, Docking of conjugates linked via ethylene and 1-oxoethylene spacers to BChE (a) Figure 4 overlapped with binding pose of dimebon (carbon atoms are shown in orange) (b) Figure 3a overlapped with binding pose of donepezil (carbon atoms are shown in bright green), viewpoint differs from the original figures in the text; Figure S4, Docking of conjugates **4** linked via ethylene spacer to AChE (a) Figure 5a overlapped with binding pose of donepezil (carbon atoms are shown in bright green) (b) Figure 5b overlapped with binding pose of donepezil (carbon atoms are shown in bright green) (c) Figure 5b overlapped with binding pose of dimebon (carbon atoms are shown in orange), viewpoint differs from the original figures in the text; Figure S5, Calculation of IC_50_ values for compounds binding with MK-801 specific site of NMDA receptor (MEAN ± SEM, *n* = 3); Figure S6, Calculation of IC_50_ values for compounds binding with ifenprodil specific site of NMDA receptor (MEAN ± SEM, *n* = 3).

## Sample Availability

Not available.

## Abbreviations

AChE	acetylcholinesterase;
BChE	butyrylcholinesterase;
BNPP	bis-4-nitrophenyl phosphate;
CAS	catalytic active site;
CES	carboxylesterase;
DMSO	dimethyl sulfoxide;
*Ee*AChE	AChE from *Electrophorus electricus*;
MPT	mitochondrial permeability transition;
PAS	peripheral anionic site;
QM/MM	quantum mechanics/molecular mechanics;
Tb+MAP	tubulin and microtubule-associated proteins.
