# Supplementary material for: Conjugation of Aminoadamantane and γ-Carboline Pharmacophores Gives Rise to Unexpected Properties of Multifunctional Ligands
Source: Molecules. 2021 Sep 11;26(18):5527. doi: 10.3390/molecules26185527 (PMC8471380; doi:10.3390/molecules26185527)
Supplement: Supplementary file 1 [file molecules-26-05527-s001.zip › molecules-1347708-supplementary.pdf]

Conjugation of Aminoadamantane and γ-Carboline Pharmacophores Gives Rise to Unexpected Properties of Multifunctional Ligands

Sergey O. Bachurin [1], Galina F. Makhaeva [1], Elena F. Shevtsova [1], Alexey Yu. Aksinenko [1], Vladimir V. Grigoriev [1], Pavel N. Shevtsov [1], Tatiana V. Goreva [1], Tatiana A. Epishina [1], Nadezhda V. Kovaleva [1], Elena A. Pushkareva [1], Natalia P. Boltneva [1], Sofya V. Lushchekina [1,2], Alexey V. Gabrelyan [1], Vladimir L. Zamoyski [1], Lyudmila G. Dubova [1], Elena V. Rudakova [1], Vladimir P. Fisenko [3], Elena V. Bovina [1] and Rudy J. Richardson [4]

[1] Institute of Physiologically Active Compounds, Russian Academy of Sciences, Chernogolovka, 142432, Russia; bachurin@ipac.ac.ru (S.O.B.); gmakh@ipac.ac.ru (G.F.M.); shevtsova@ipac.ac.ru (E.F.S.); alaks@ipac.ac.ru (A.Yu.A.); grigor@ipac.ac.ru (V.V.G.); pnshevtsov@gmail.com (P.N.S.); tg1960@mail.ru (T.V.G.); tatiana.epishina@mail.ru (T.A.E.); kovalevanv@ipac.ac.ru (N.V.K.); l.kickeeva@yandex.ru (E.A.P.); boltneva@ipac.ac.ru (N.P.B.); sofya.lushchekina@gmail.com (S.V.L.); avg@ipac.ac.ru (A.V.G.), vzam@yandex.ru (V.L.Z.); dubova@ipac.ac.ru (L.G.D.); rudakova@ipac.ac.ru (E.V.R.); bovina_e@ipac.ac.ru (E.V.B.)

[2] Emanuel Institute of Biochemical Physics, Russian Academy of Sciences, Moscow, 119334, Russia; sofya.lushchekina@gmail.com (S.V.L.)

[3] I.M. Sechenov First Moscow State Medical University, 8 build. 2 Trubetskaya str., Moscow 119991, Russia; vpfisenko@mail.ru (V.P.F)

[4] Department of Environmental Health Sciences, University of Michigan, Ann Arbor, MI 48109 USA; rjrich@umich.edu (R.J.R.)

* Correspondence: bachurin@ipac.ac.ru; Tel.: +7(496)524-2650

Contents

Table S1. Docking scores of aminoadamantane–γ-carboline conjugates.

Figure S1. IC_{50} values for hAChE inhibition by compounds **4** (MEAN ± SEM, n = 3).

Figure S2. IC_{50} values for eqBChE inhibition by compounds **4, 8** (MEAN ± SEM, n = 3).

Figure S3. Docking of conjugates linked *via* ethylene and 1-oxoethylene spacers to BChE (a) Fig. 4 overlapped with binding pose of dimebon (carbon atoms are shown in orange) (b) Fig 3a overlapped with binding pose of donepezil (carbon atoms are shown in bright green), viewpoint differs from the original figures in the text.

Figure S4. Docking of conjugates **4** linked *via* ethylene spacer to AChE (a) Fig. 5a overlapped with binding pose of donepezil (carbon atoms are shown in bright green) (b) Fig. 5b overlapped with binding pose of donepezil (carbon atoms are shown in bright green) (c) Fig. 5b overlapped with binding pose of dimebon (carbon atoms are shown in orange), viewpoint differs from the original figures in the text.

Figure S5.Representative curves for calculation of IC_{50} values for compounds binding with MK-801 specific site of NMDA receptor in semilogarithmic plotes (MEAN ± SEM, n = 3).

Figure S6. Representative curves for calculation of IC_{50} values for compounds binding with ifenprodil specific site of NMDA receptor in semilogarithmic plotes (MEAN ± SEM, n = 3).

Supplementary

Table S1. Docking scores of aminoadamantane–γ-carboline conjugates.

No	R	Estimated docked binding energies (kcal/mol)	
		**AChE**	**BChE**
**4a**	H	–9.80	–11.25
**4b**	Me	–10.47	–10.21
**4c**	F	–10.10	–11.01
**8a**	H	–9.72	–10.66
**8b**	Me	–9.53	–10.36
**8c**	F	–9.49	–10.22
**4d**	H	–10.38	–10.75
**4e**	Me	–10.46	–10.64
**4f**	F	–10.68	–10.70
**8d**	H	–8.94	–10.60
**8e**	Me	–9.33	–10.09
**8f**	F	–9.72	–10.60
**dimebon**		–9.98	–10.35
**donepezil**		–11.66	–10.42

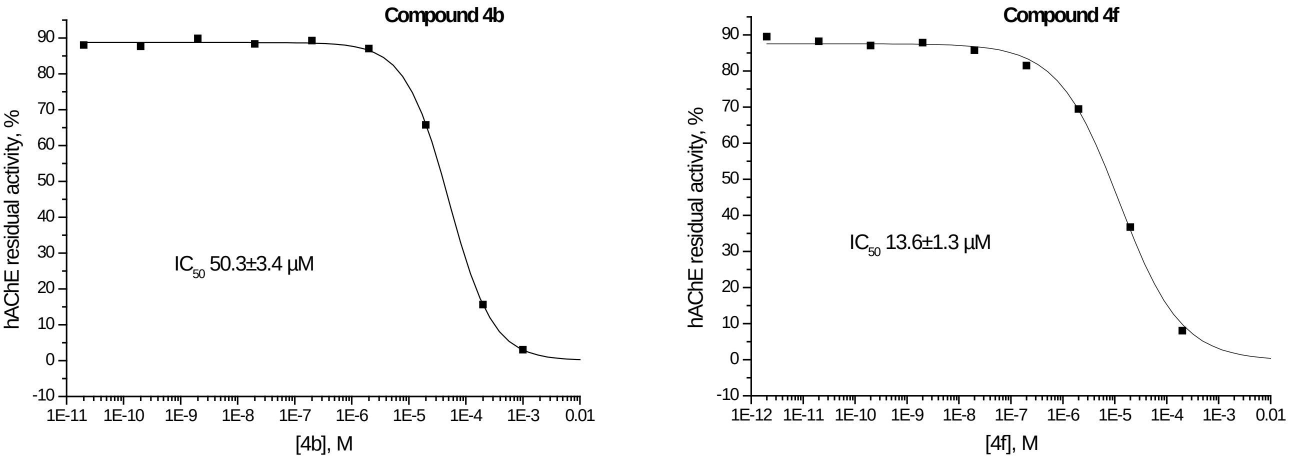

Figure S1. IC_{50} values for hAChE inhibition by compounds **4** (MEAN ± SEM, n = 3).

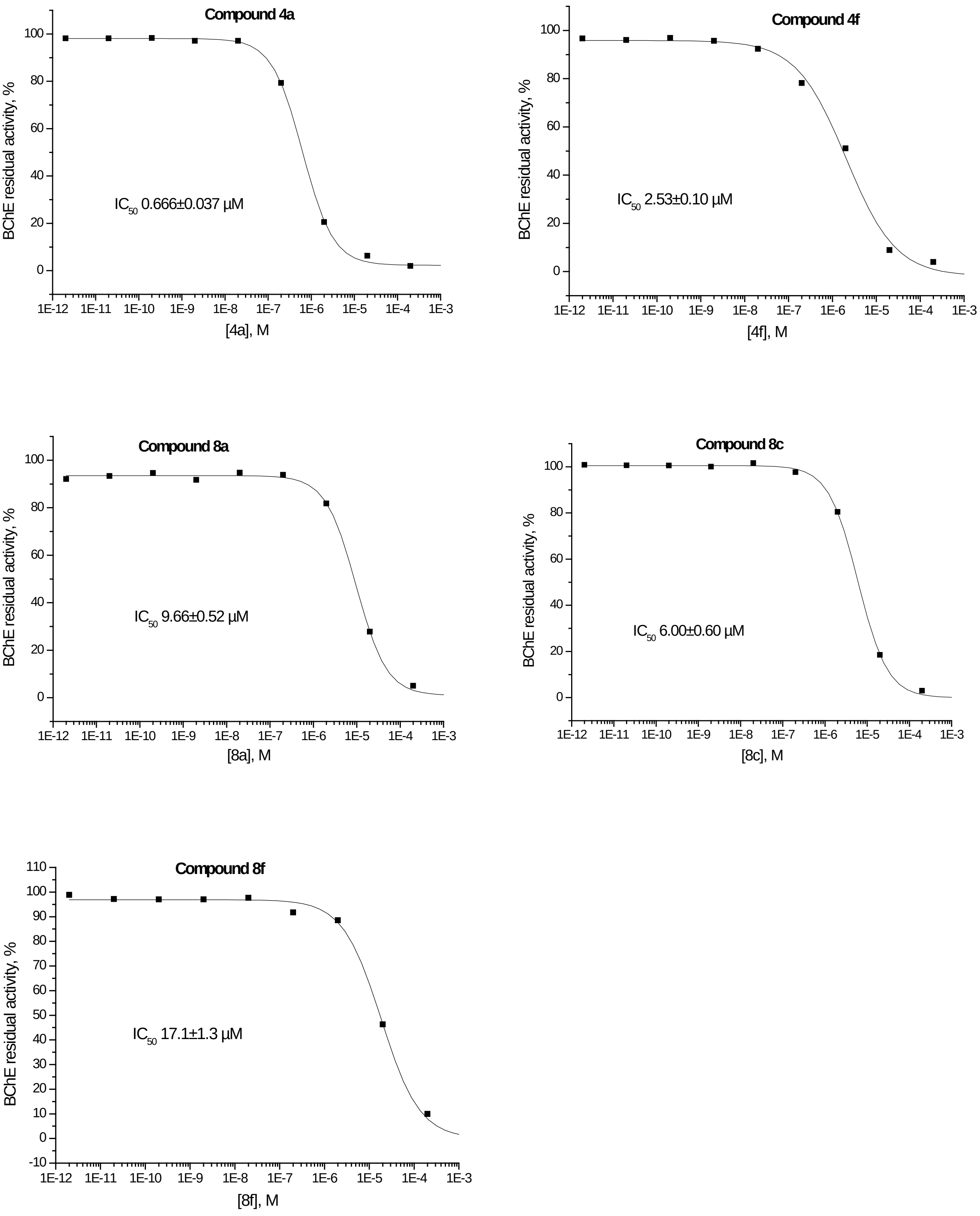

Figure S2. IC_{50} values for eqBChE inhibition by compounds **4, 8** (MEAN ± SEM, n = 3).

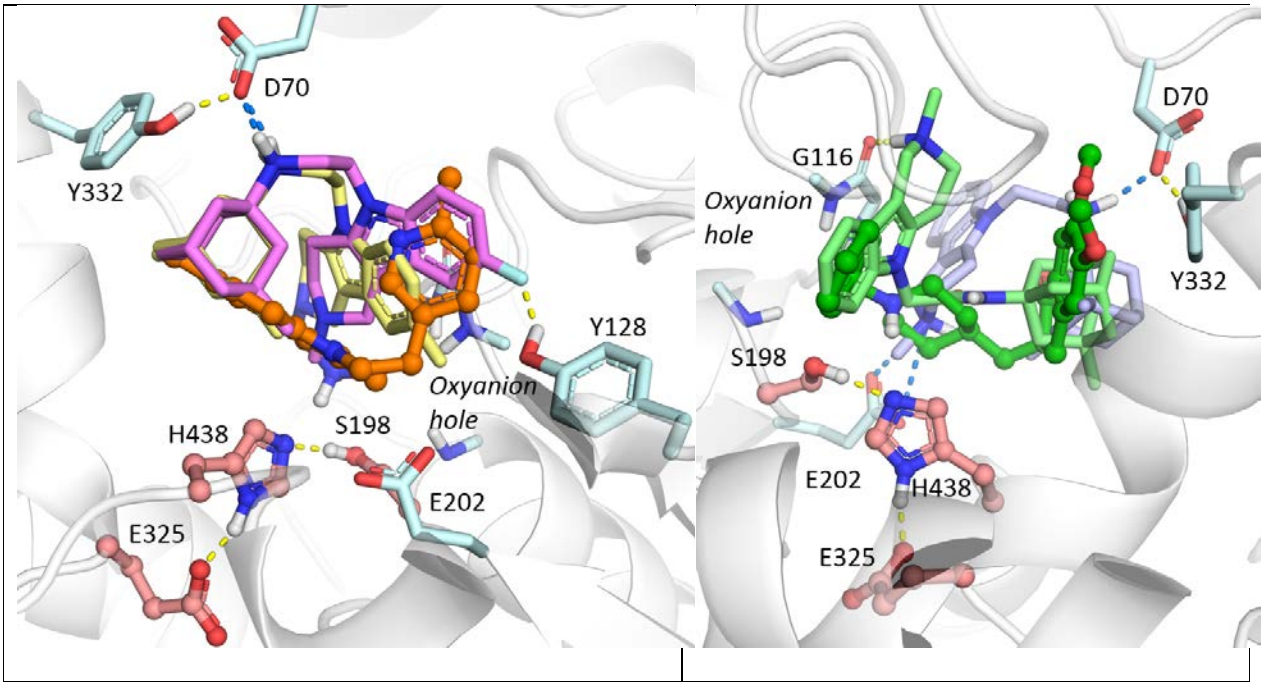

(**a**) (**b**)

Figure S3. Docking of conjugates linked *via* ethylene and 1-oxoethylene spacers to BChE (a) Fig. 4 overlapped with binding pose of dimebon (carbon atoms are shown in orange) (b) Fig 3a overlapped with binding pose of donepezil (carbon atoms are shown in bright green), viewpoint differs from the original figures in the text.

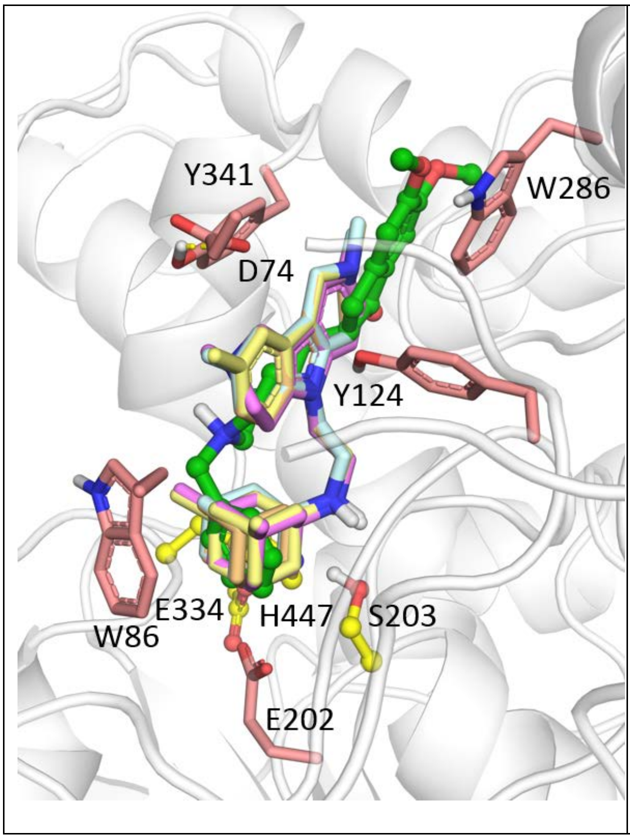

(**a**)

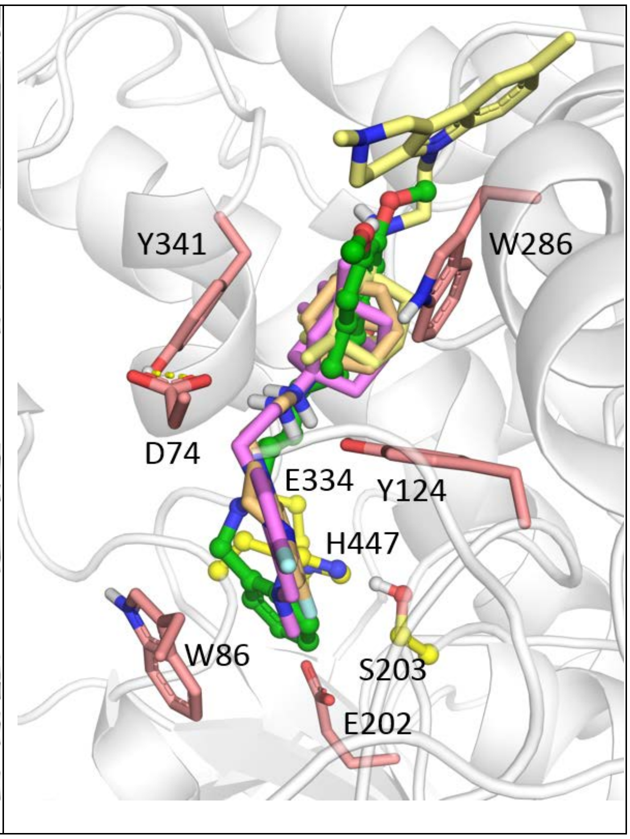

(**b**)

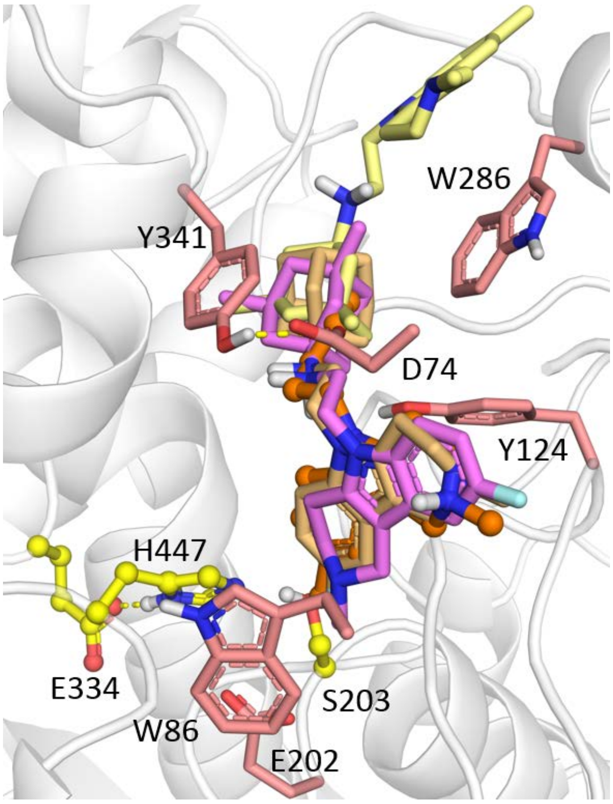

(**c**)

Figure S4. Docking of conjugates **4** linked *via* ethylene spacer to AChE (a) Fig. 5a overlapped with binding pose of donepezil (carbon atoms are shown in bright green) (b) Fig. 5b overlapped with binding pose of donepezil (carbon atoms are shown in bright green) (c) Fig. 5b overlapped with binding pose of dimebon (carbon atoms are shown in orange), viewpoint differs from the original figures in the text.

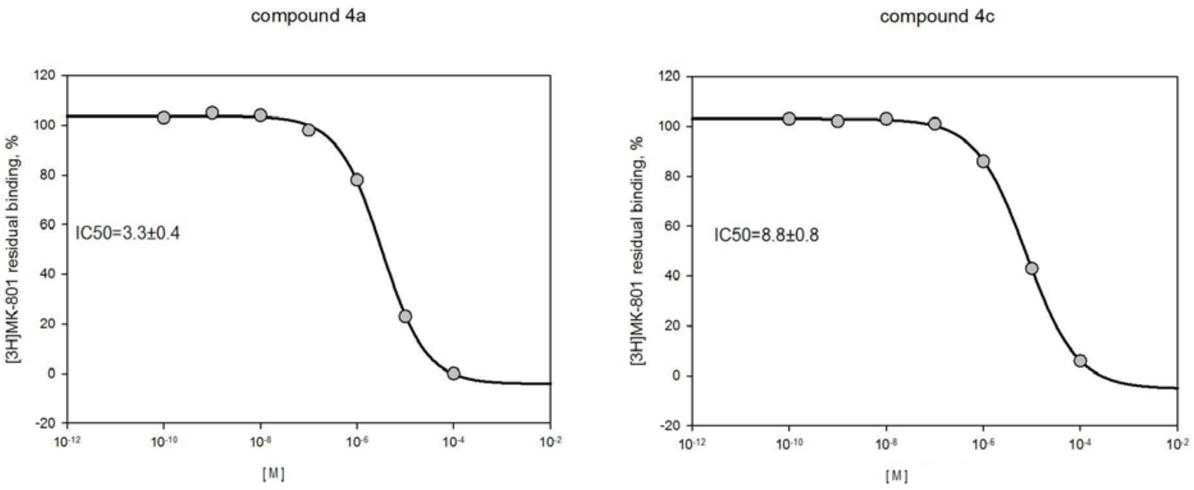

Figure S5. Representative curves for calculation of IC_{50} values for compounds binding with MK-801 specific site of NMDA receptor in semilogarithmic plotes (MEAN ± SEM, n = 3).

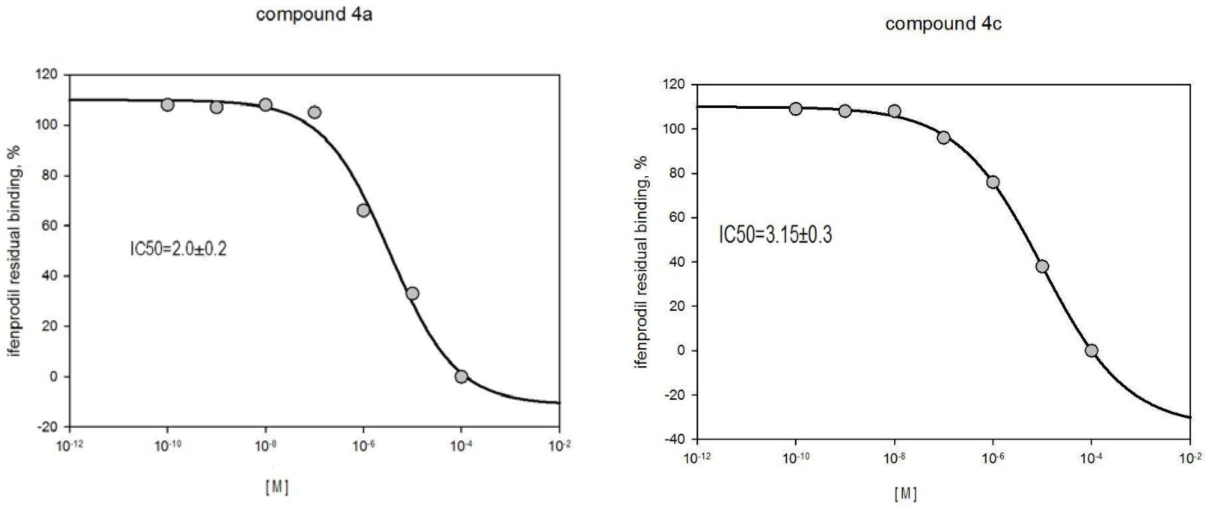

Figure S6. Representative curves for calculation of IC_{50} values for compounds binding with ifenprodil specific site of NMDA receptor in semilogarithmic plotes (MEAN ± SEM, n = 3).
